# Supplementary material for: Ortho-Functionalized Dibenzhydryl Substituents in α-Diimine Pd Catalyzed Ethylene Polymerization and Copolymerization
Source: Polymers (Basel). 2020 Oct 28;12(11):2509. doi: 10.3390/polym12112509 (PMC7692462; doi:10.3390/polym12112509)
Supplement: Supplementary file 1 [file polymers-12-02509-s001.pdf]

Ortho-functionalized dibenzhydryl substituents in α-diimine Pd catalyzed ethylene polymerization and copolymerization

Qasim Muhammad [1], Wenmin Pang [1], Fuzhou Wang [2,*] and Chen Tan [2,*]

[1] Department of Polymer Science and Engineering, University of Science and Technology of China, Hefei, China;
[2] Institutes of Physical Science and Information Technology, Key Laboratory of Structure and Functional Regulation of Hybrid Materials of Ministry of Education, Anhui University, Hefei, China.
* Correspondence: wangfuzhou@ahu.edu.cn (F.W.); ctan@ahu.edu.cn (C.T.)

Content

1. **NMR Spectra of the Amines, Ligands and Catalysts 2**

2. **MS Spectra of the Amines, Ligands and Catalysts 18**

3. **NMR Spectra of the Polymers 24**

4. **GPC Results of the Polymers 32**

5. **X-Ray Crystallography of the Palladium Catalysts 48**

1. NMR Spectra of the Amines, Ligands and Catalysts

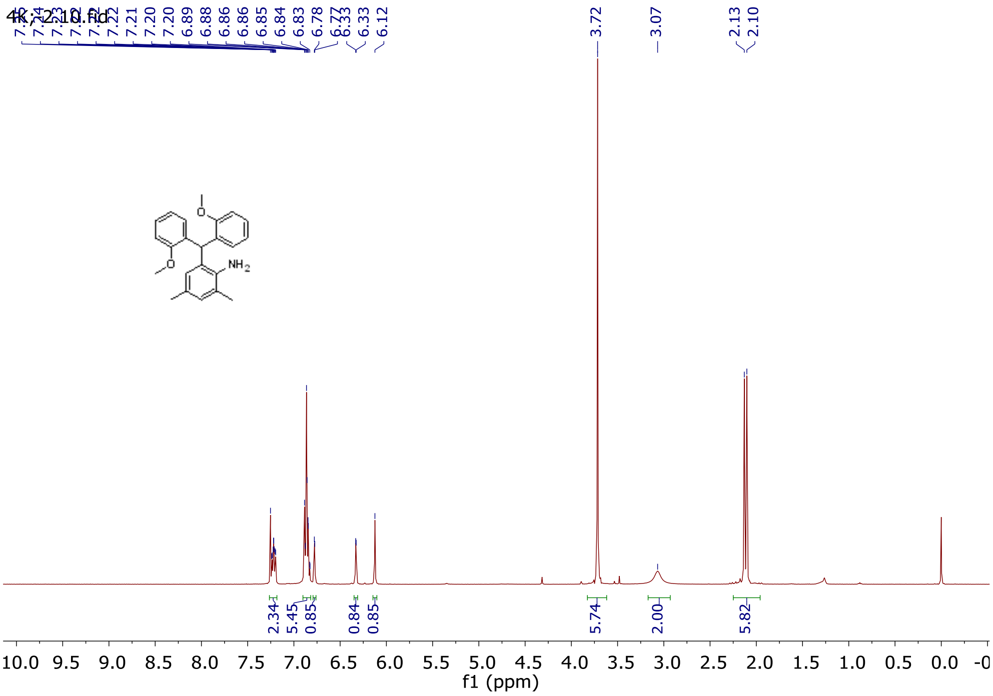

Figure S1. ^{1}H **NMR spectrum of compound 2 in** $CDCl_3$**.**

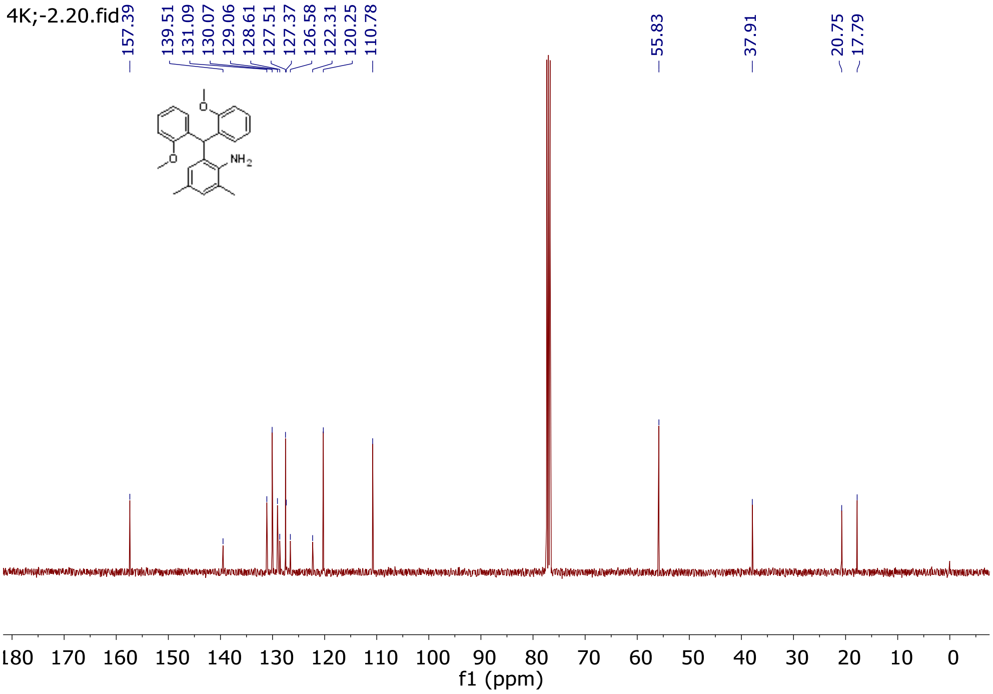

Figure S2. ^{13}C **NMR spectrum of compound 2 in** $CDCl_3$

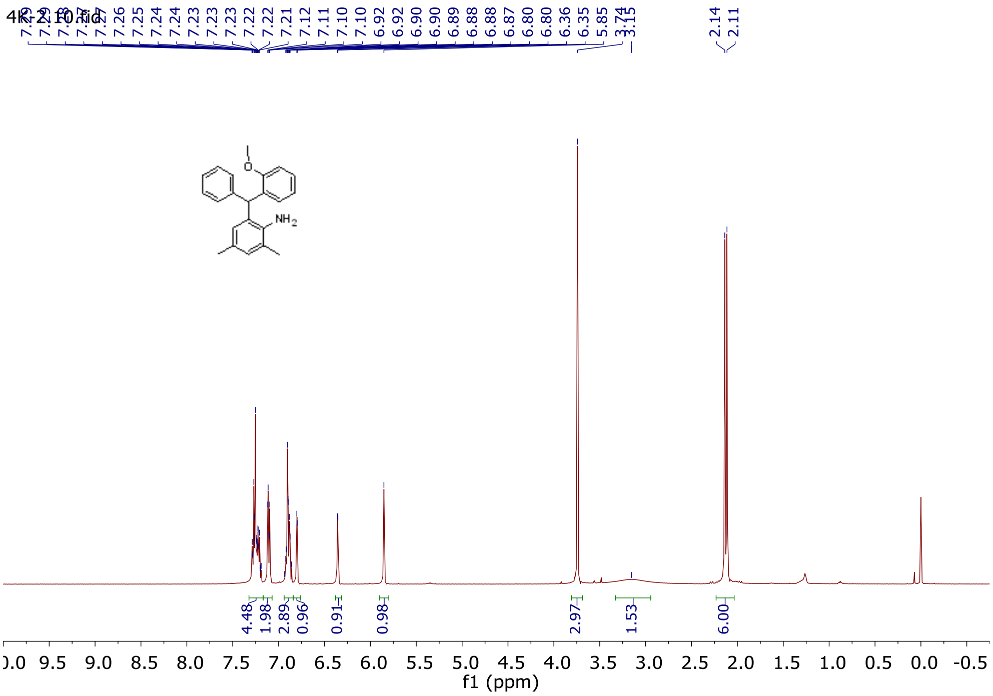

Figure S3. ^{1}H NMR spectrum of compound 3 in $CDCl_3$.

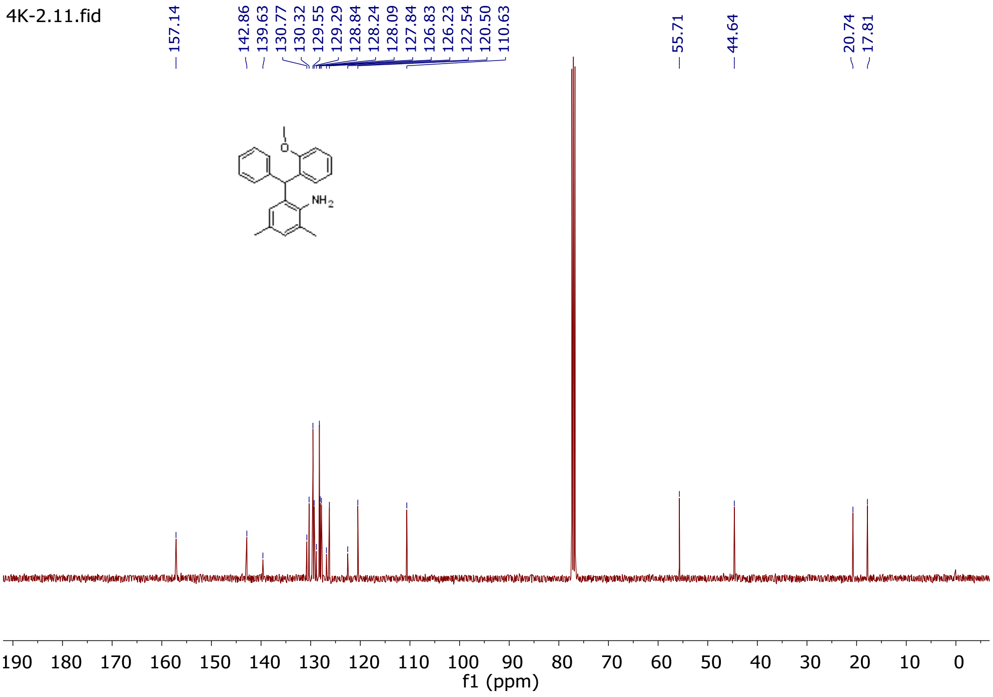

Figure S4. ^{13}C NMR spectrum of compound 3 in $CDCl_3$

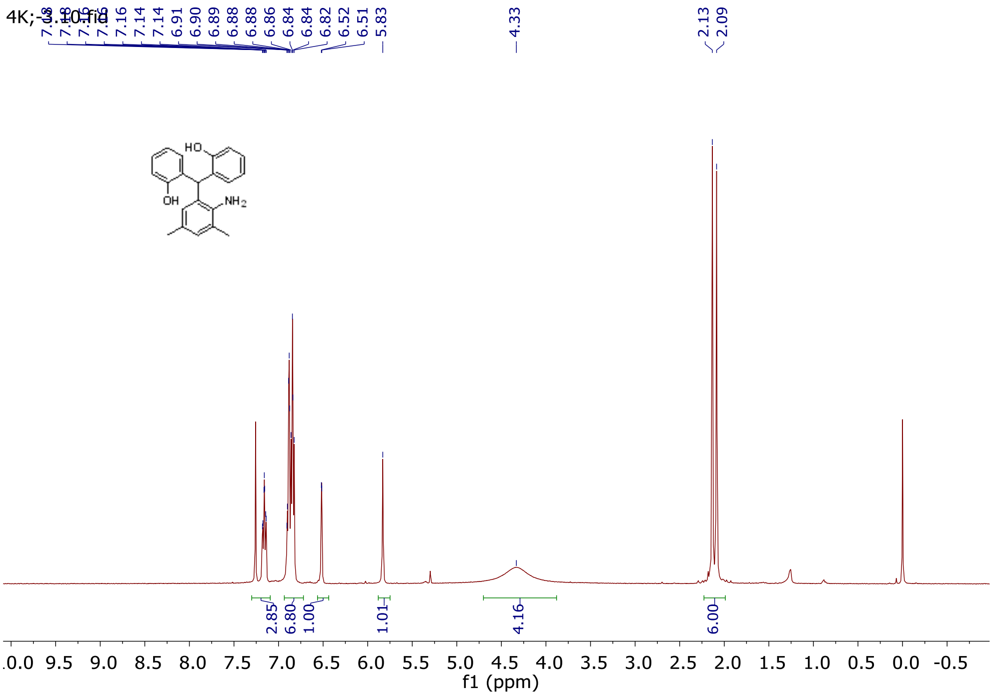

Figure S5. ^1H NMR spectrum of compound 4 in $CDCl_3$.

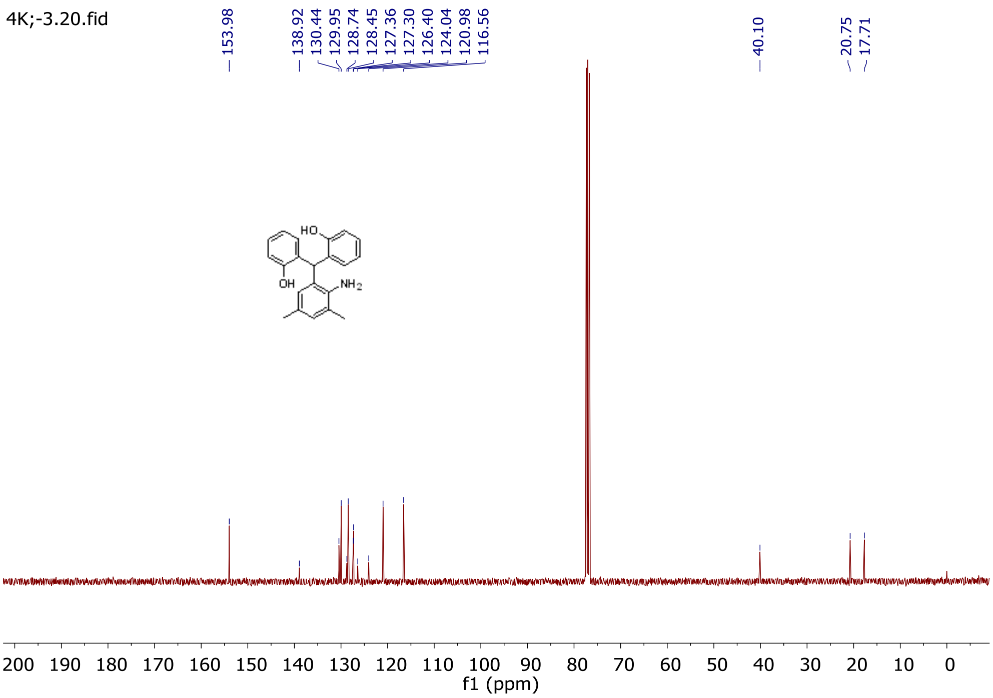

Figure S6. ^{13}C NMR spectrum of compound 4 in $CDCl_3$

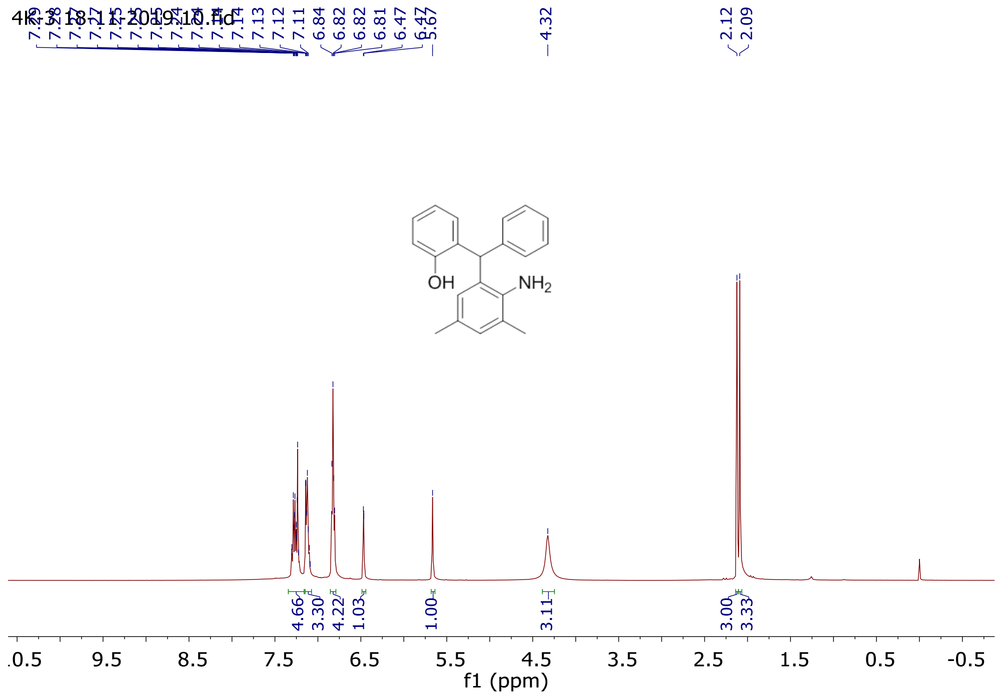

Figure S7. ^{1}**H NMR spectrum of compound 5 in** $CDCl_3$**.**

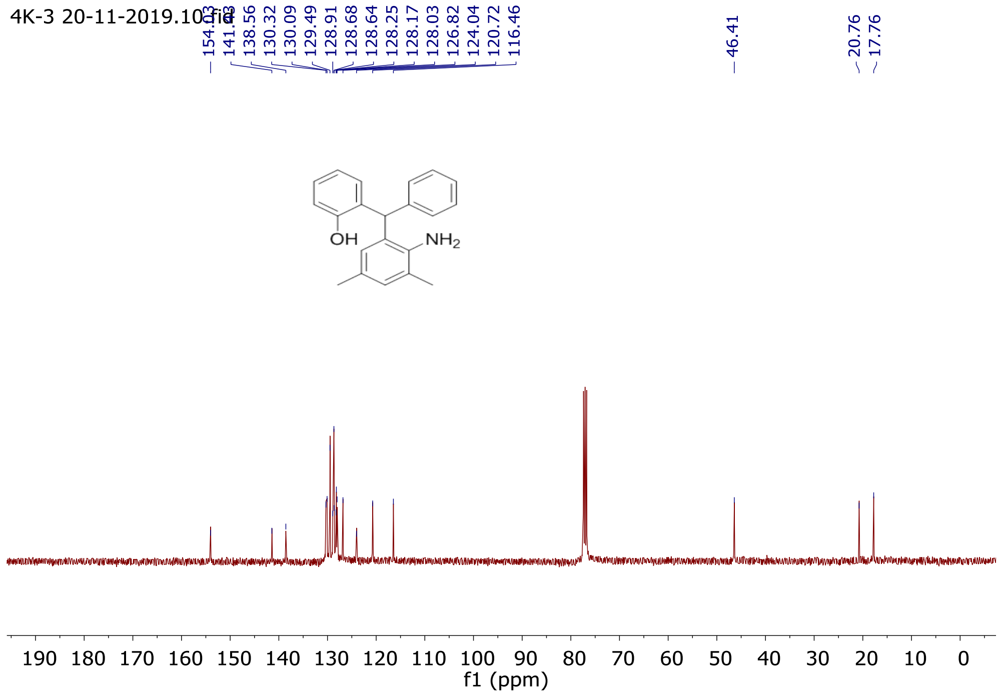

Figure S8. ^{13}**C NMR spectrum of compound 5 in** $CDCl_3$

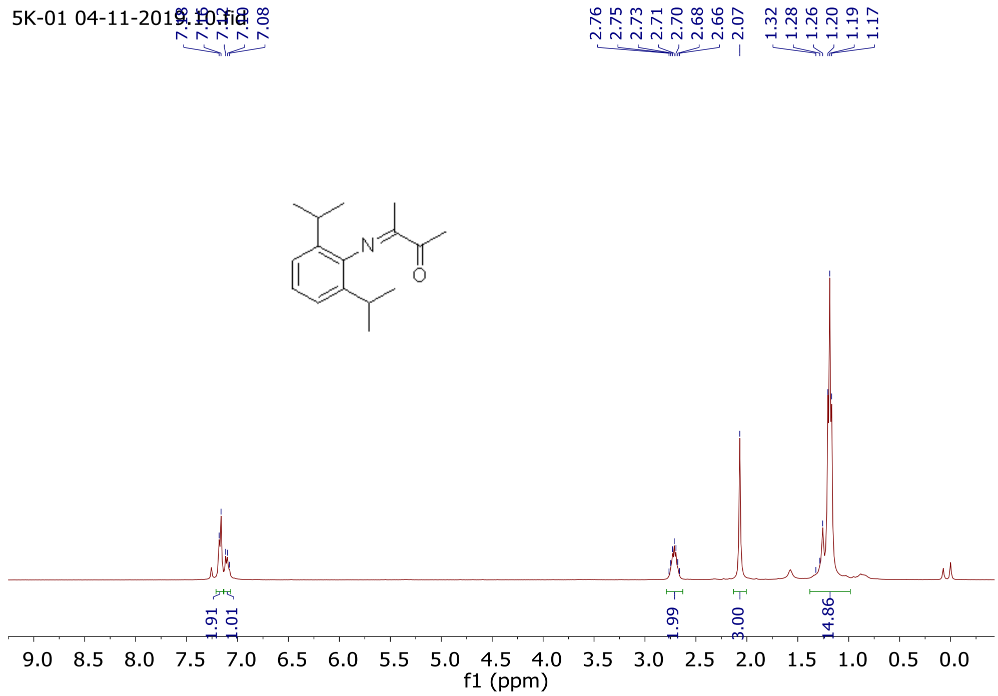

Figure S9. ^{1}H NMR spectrum of compound 6 in $CDCl_3$.

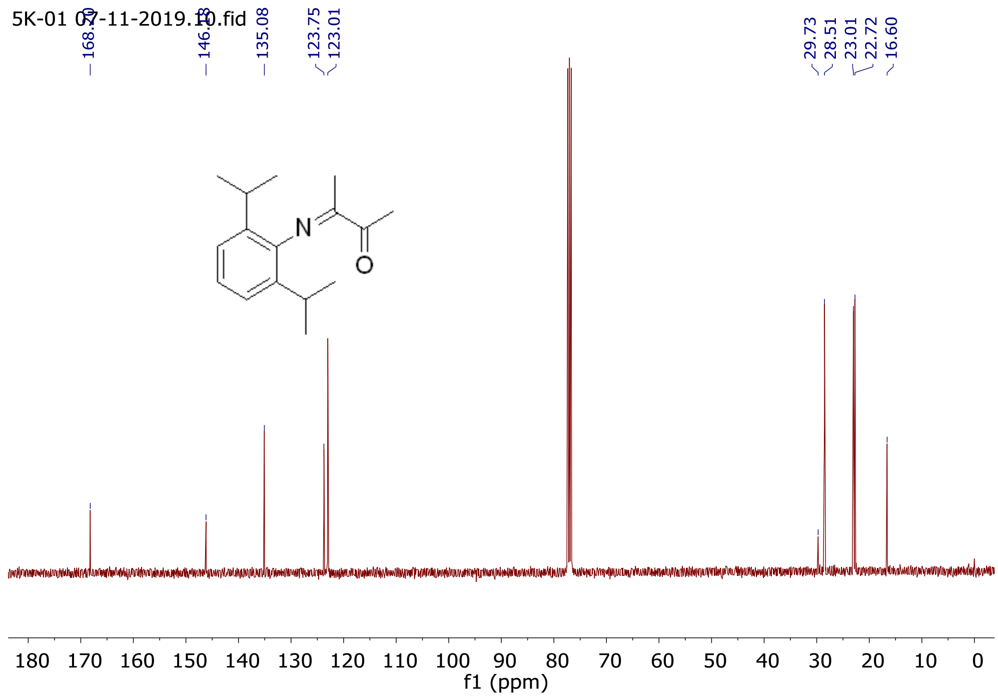

Figure S10. ^{13}C NMR spectrum of compound 6 in $CDCl_3$

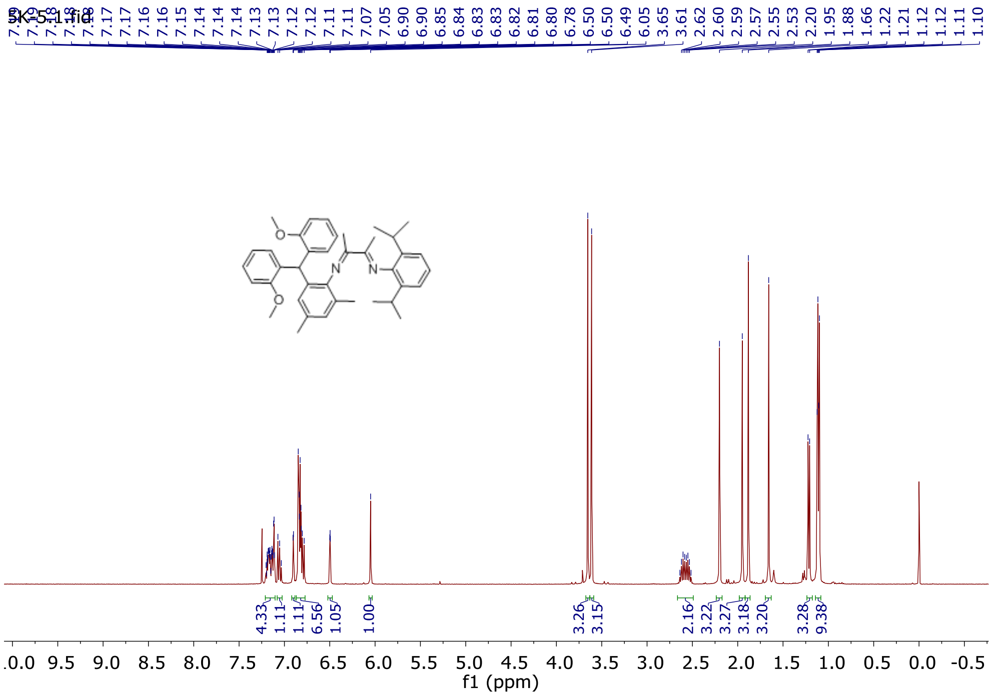

Figure S11. **^{1}H NMR spectrum of compound L1 in $CDCl_3$.**

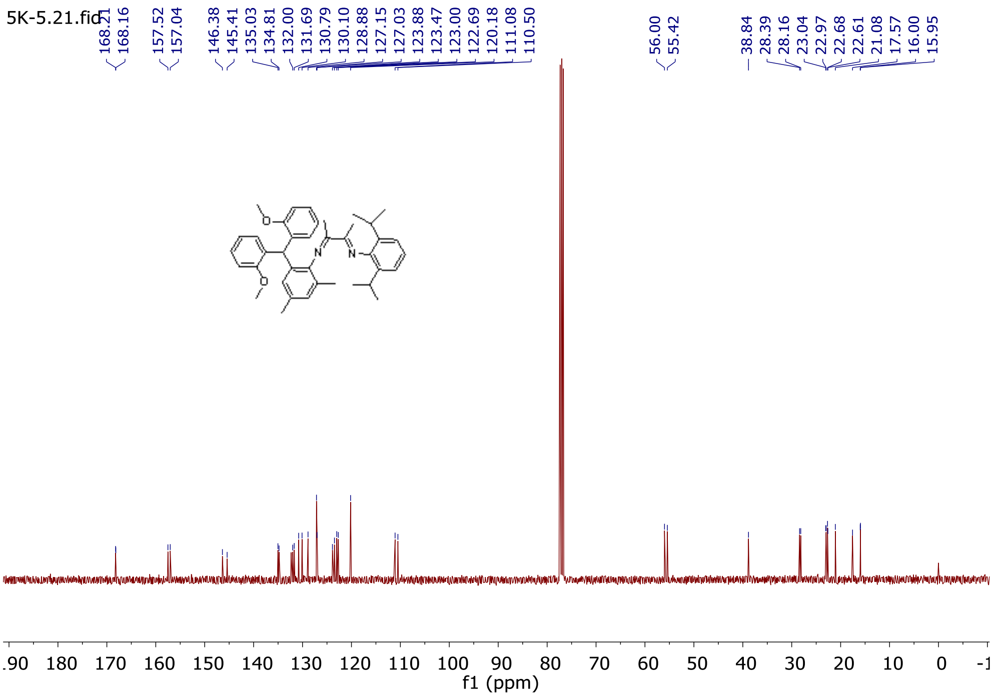

Figure S12. **^{13}C NMR spectrum of compound L1 in $CDCl_3$**

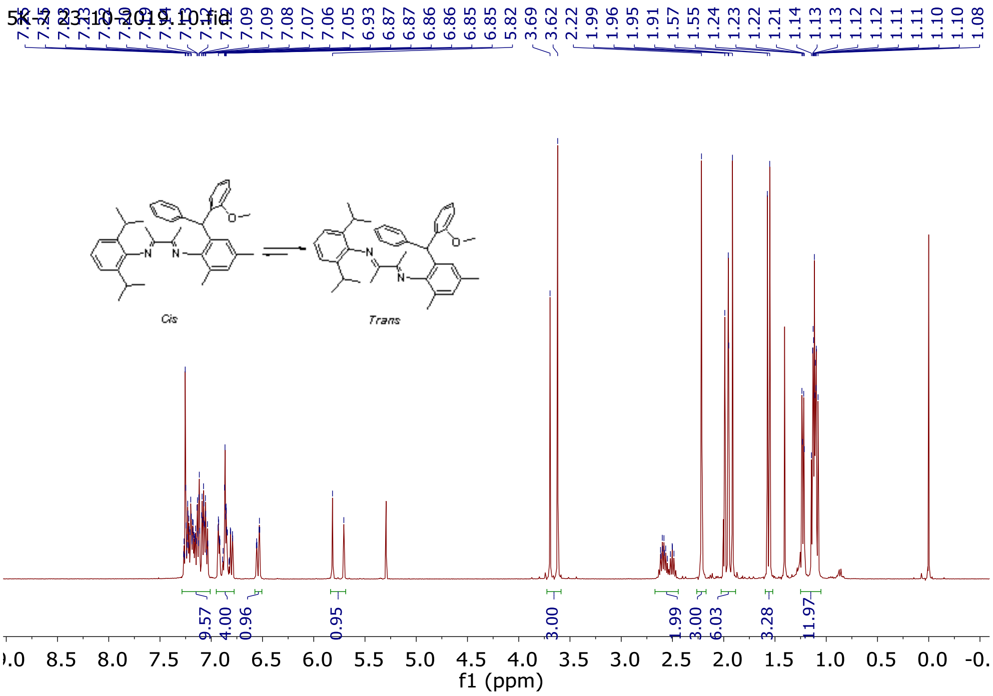

Figure S13. ^{1}H **NMR spectrum of compound L2 in** $CDCl_3$**.**

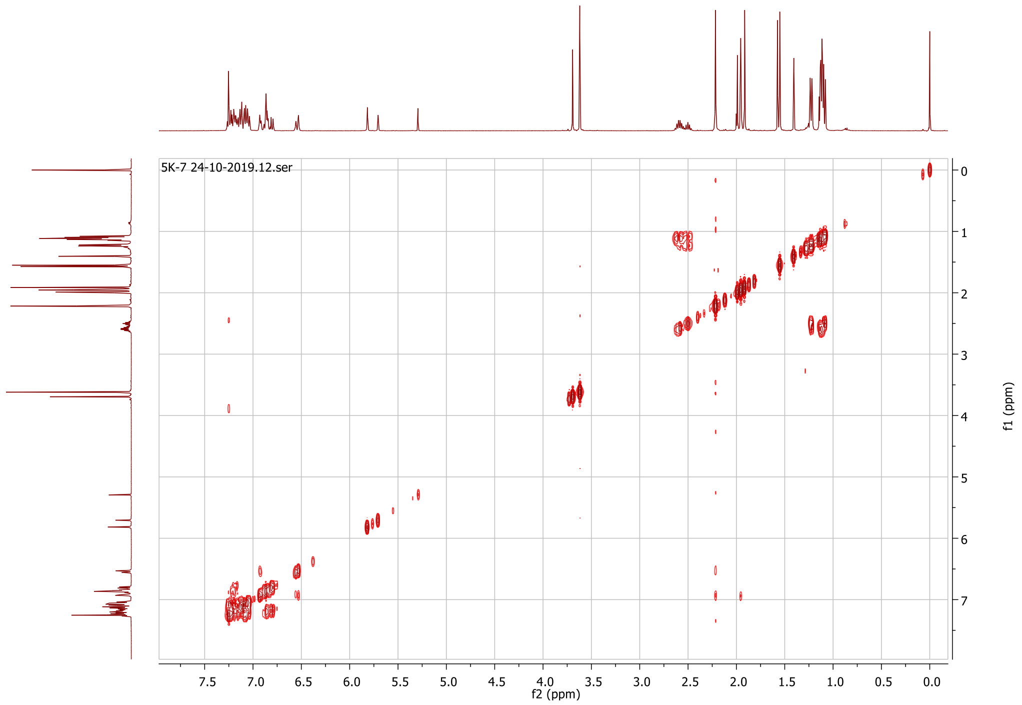

Figure S14. ^{1}H-^{1}H **COSY spectrum of compound L2 in** $CDCl_3$**.**

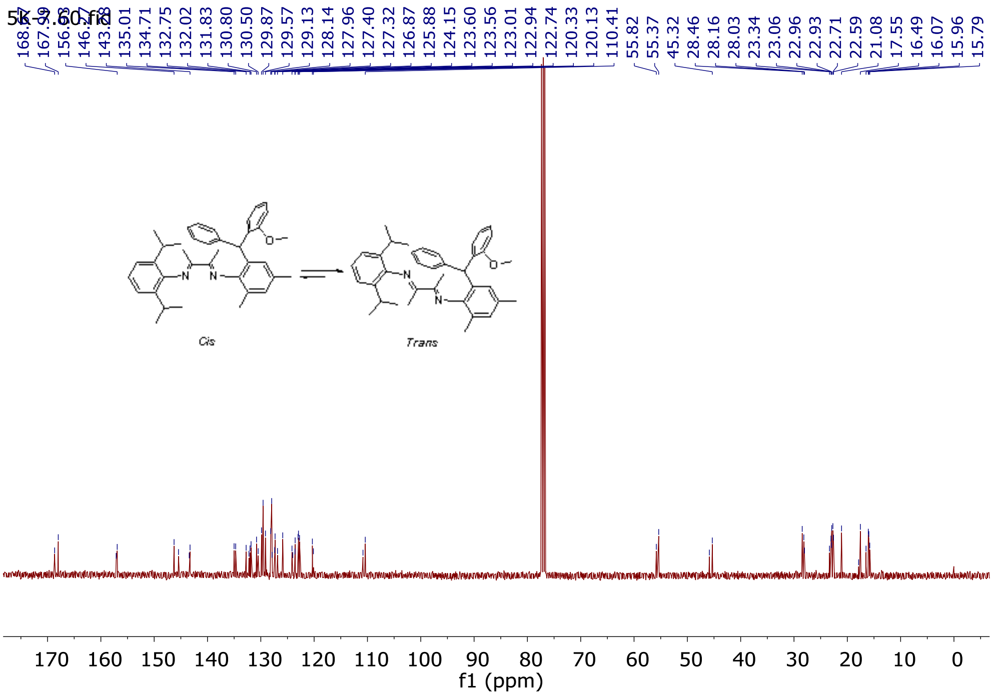

Figure S15. ^{13}C NMR spectrum of compound L2 in $CDCl_3$

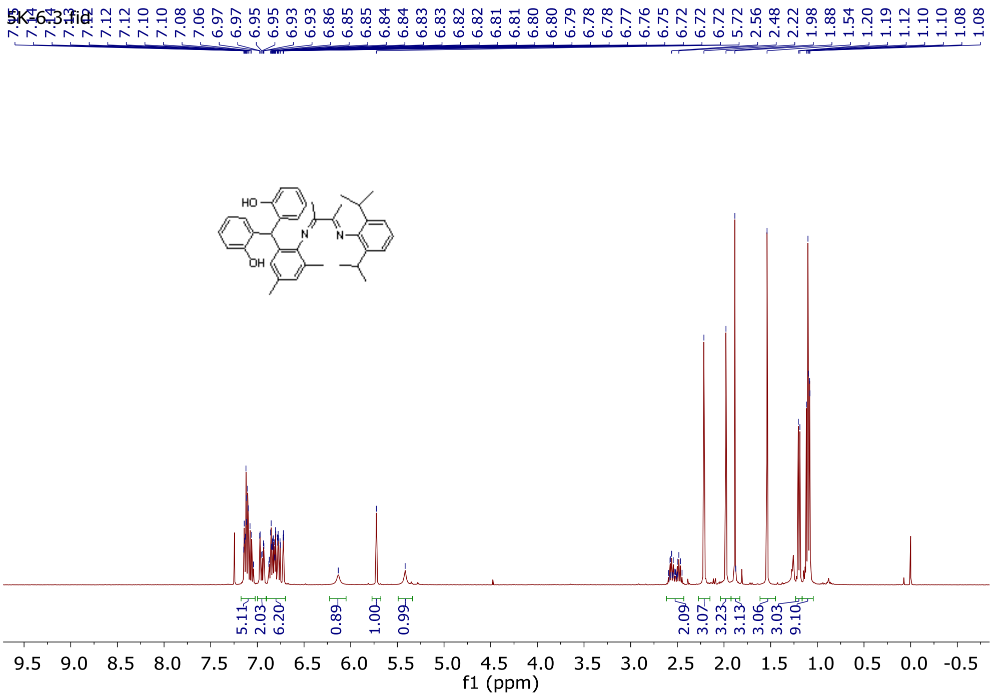

Figure S16. ^{1}H NMR spectrum of compound L3 in $CDCl_3$.

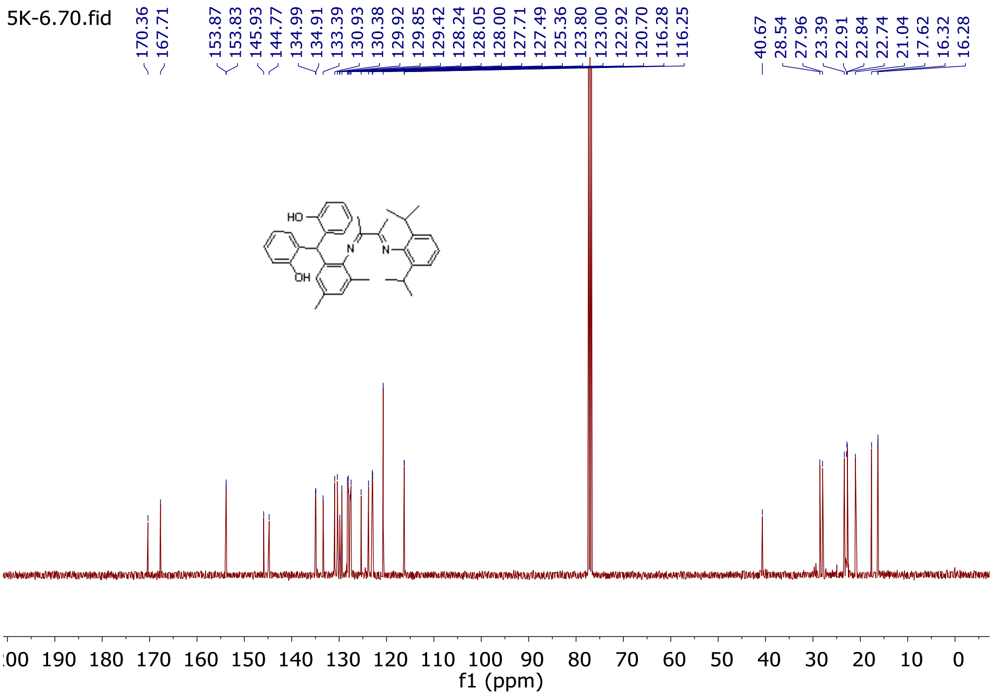

Figure S17. ^{13}**C NMR spectrum of compound L3 in CDCl$_3$**

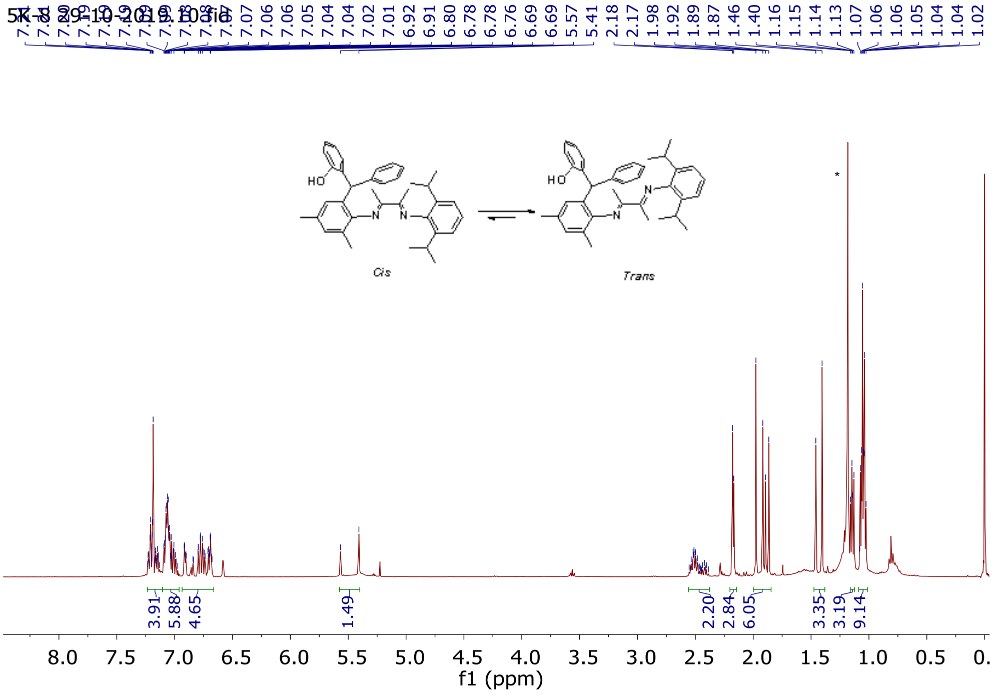

Figure S18. ^{1}**H NMR spectrum of compound L4 in CDCl$_3$.**

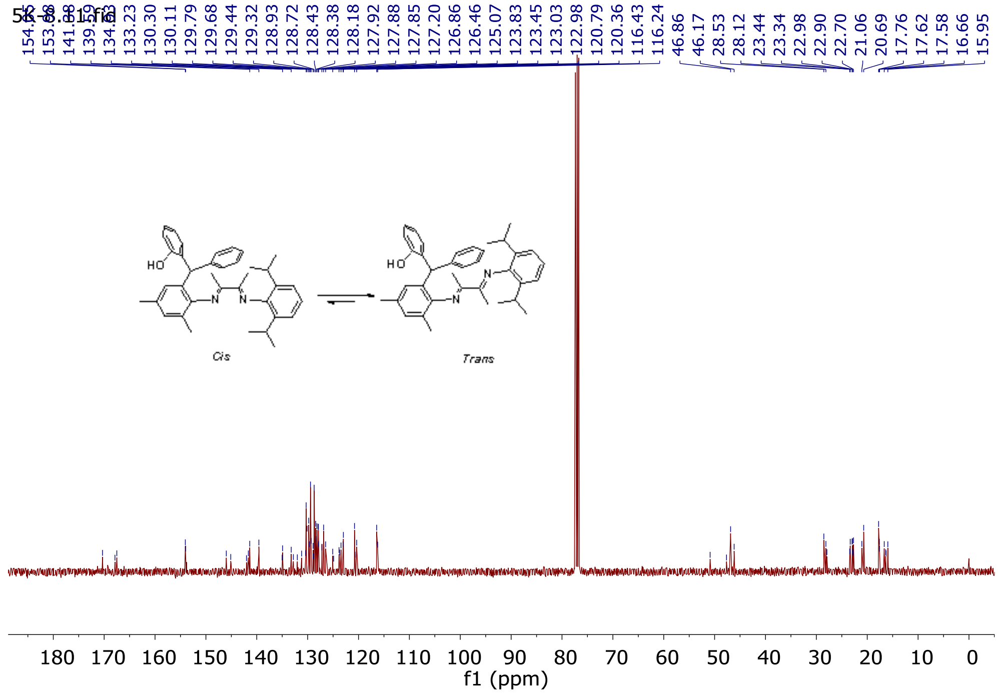

Figure S19. ^{13}C NMR spectrum of compound L4 in CDCl₃

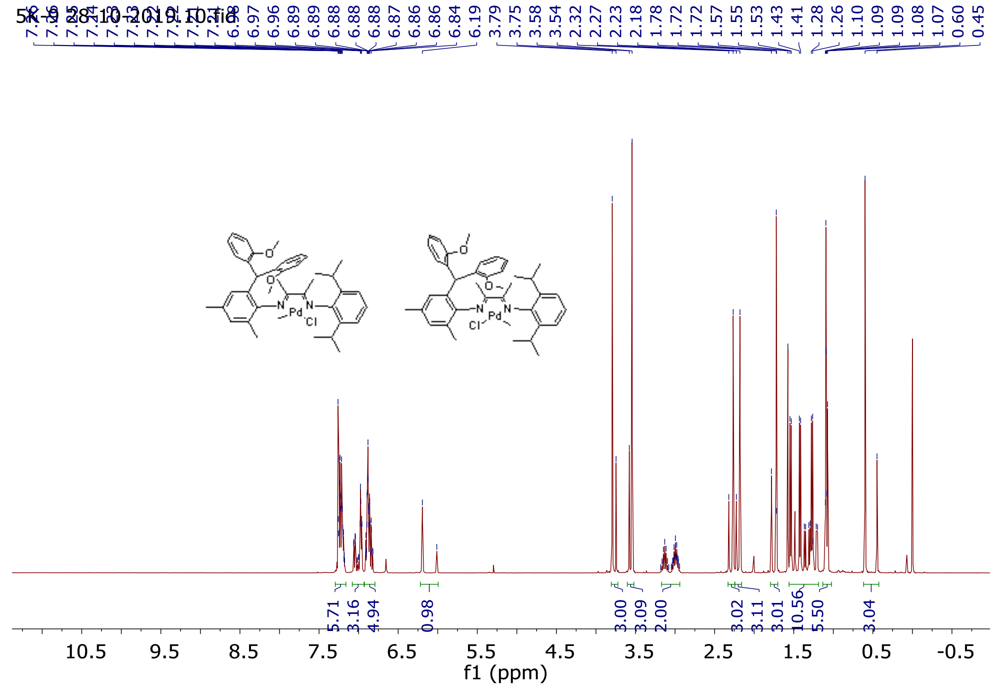

Figure S20. ^{1}H NMR spectrum of compound Pd1 in CDCl₃.

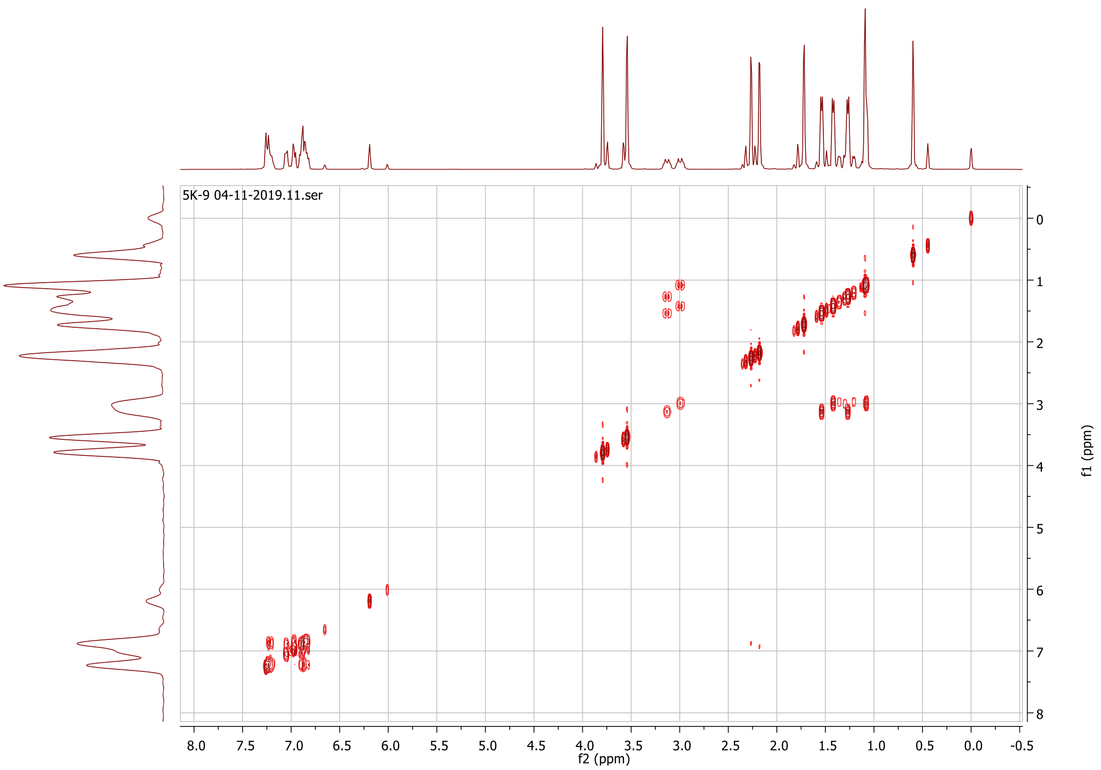

Figure S21. **¹H-¹H COSY spectrum of compound Pd1 in $CDCl_3$.**

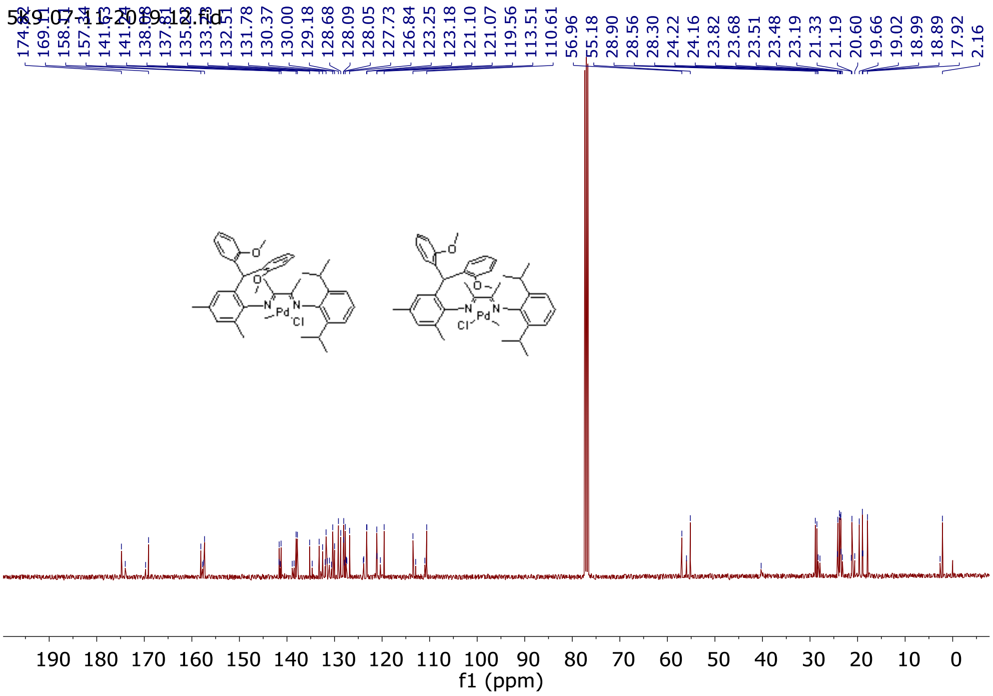

Figure S22. **¹³C NMR spectrum of compound Pd1 in $CDCl_3$**

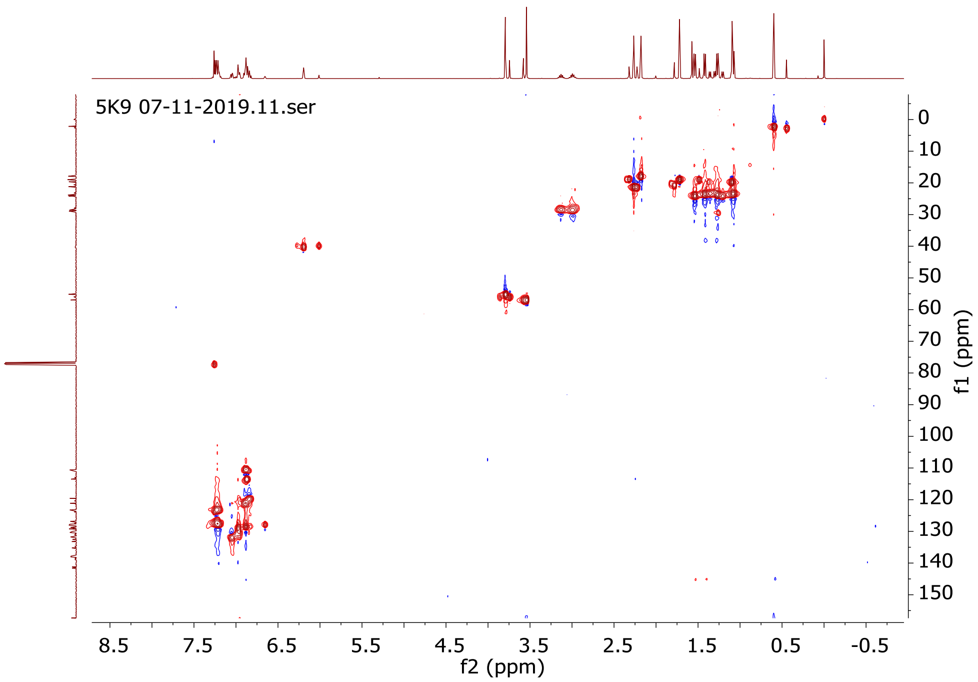

Figure S23. ^1H-^{13}C HSQC NMR spectrum of compound Pd1 in $CDCl_3$

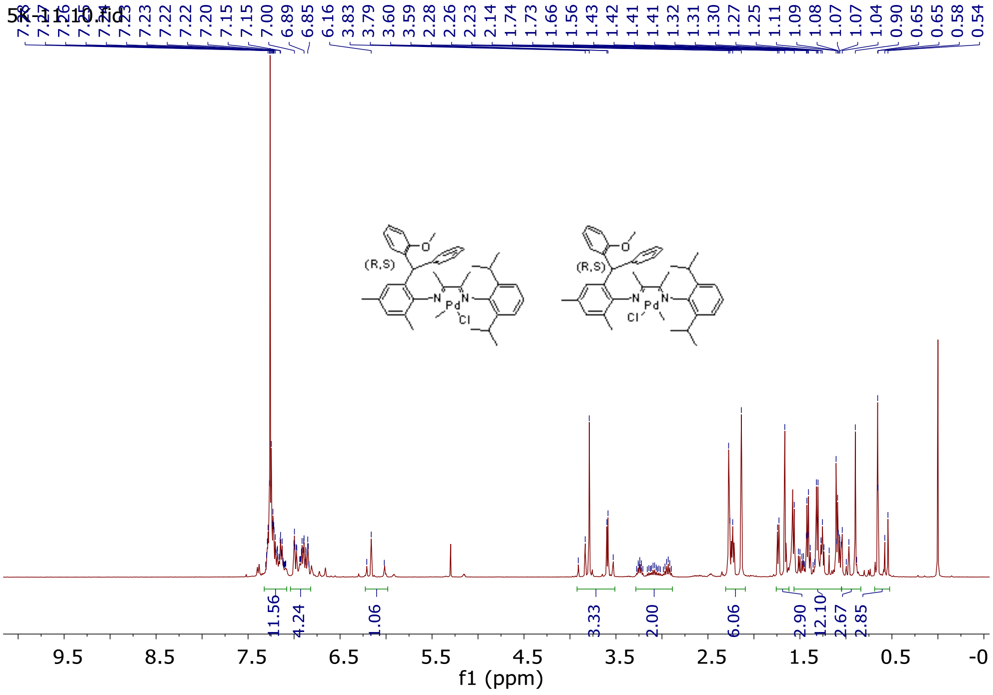

Figure S24. ^1H NMR spectrum of compound Pd2 in $CDCl_3$

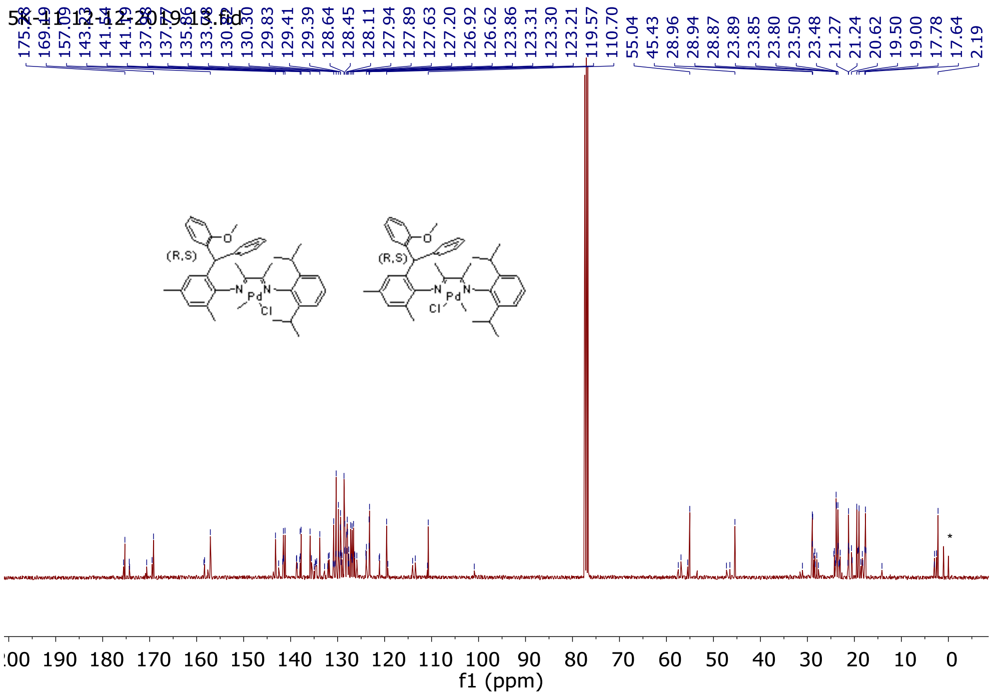

Figure S25. ^{13}C **NMR spectrum of compound Pd2 in CDCl$_3$**

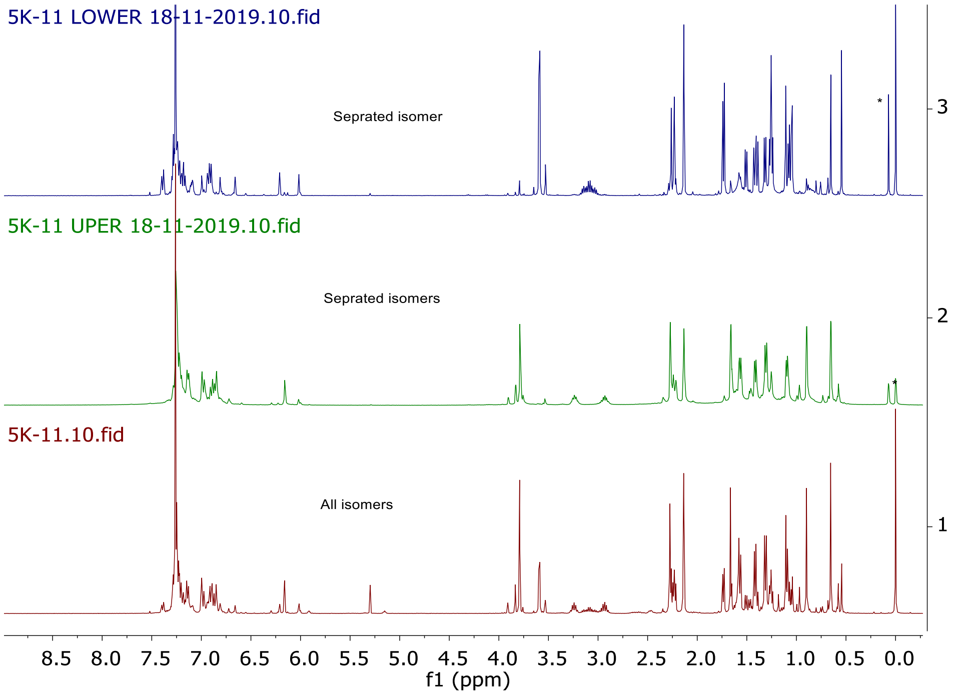

Figure S26. Comparitive ^{1}H **NMR spectra of different isomers of Pd2 in CDCl$_3$**

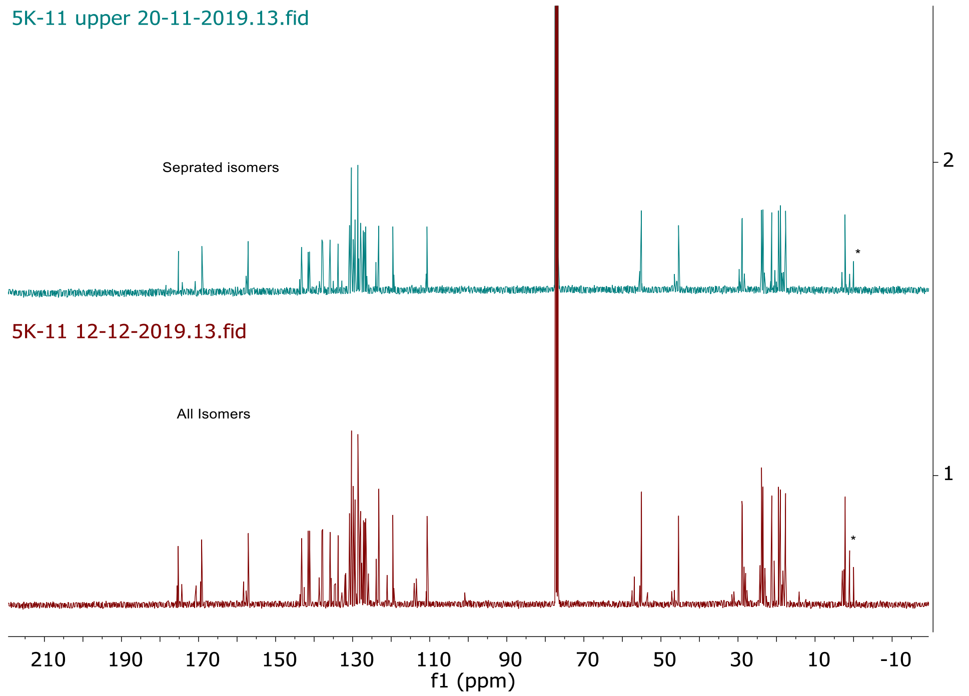

Figure S27. Comparitive ^{13}C COSY NMR spectra of different isomers of Pd2 in $CDCl_3$

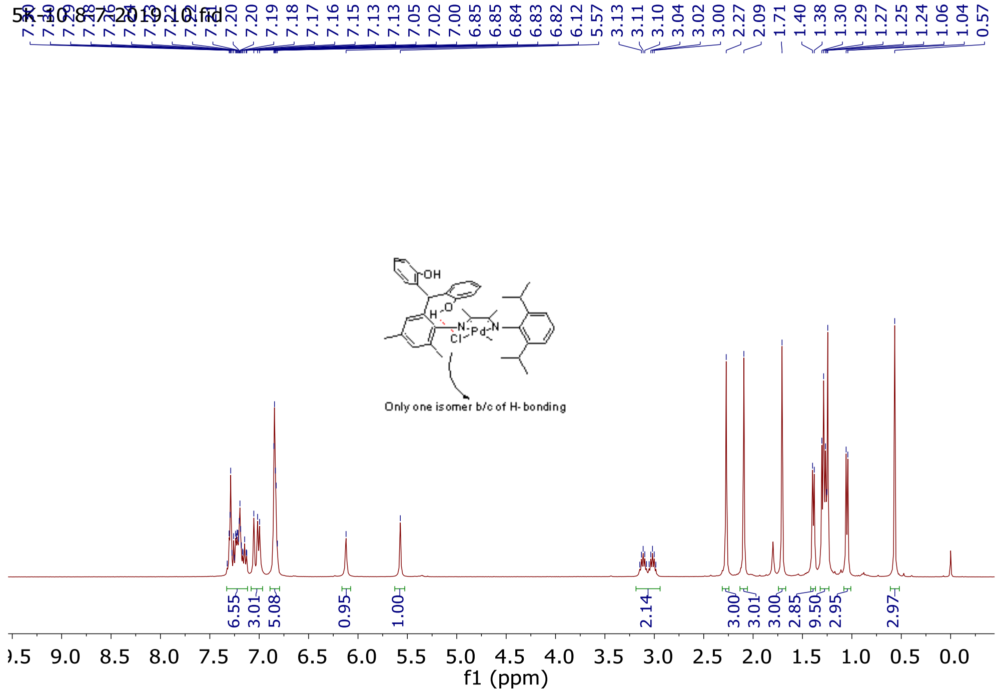

Figure S28. ^{1}H NMR spectrum of compound Pd3 in $CDCl_3$

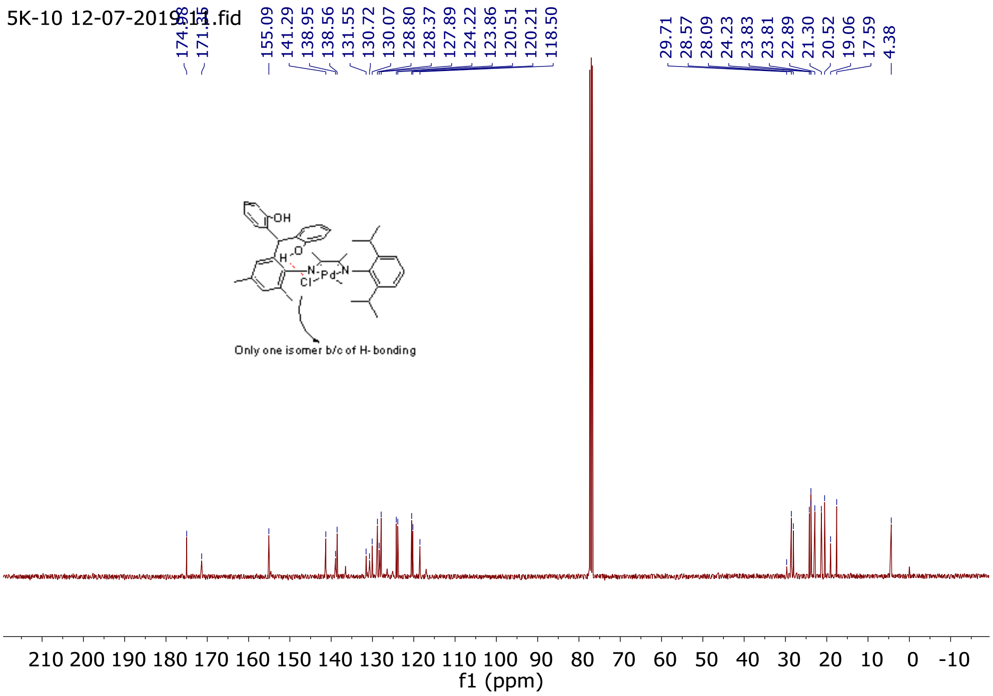

Figure S29. ^{13}C **NMR spectrum of compound Pd3 in** $CDCl_3$

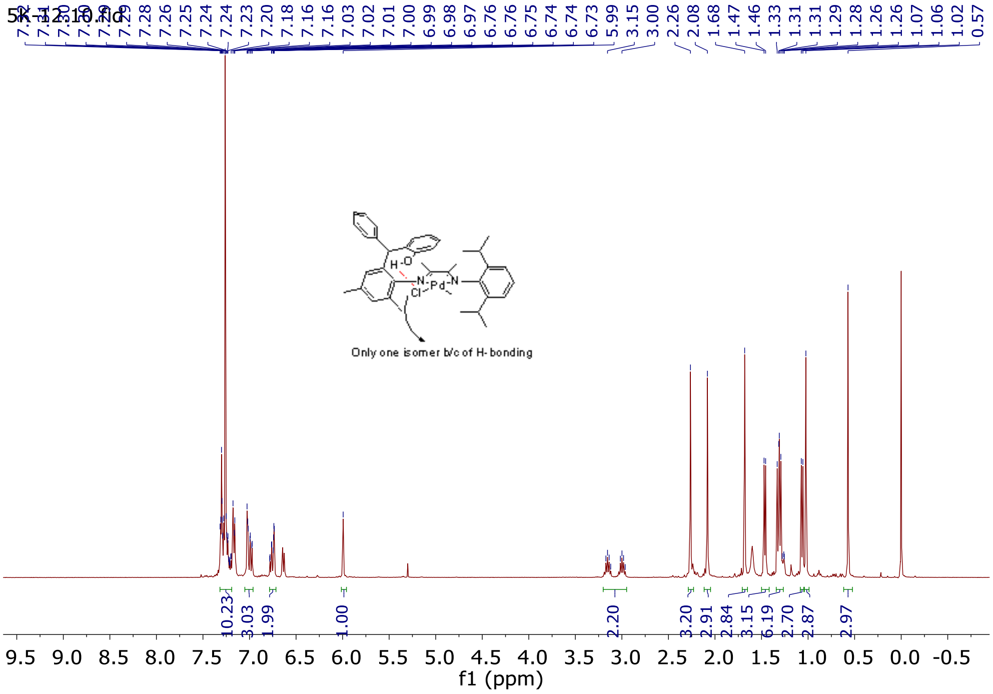

Figure S30. ^{1}H **NMR spectrum of compound Pd4 in** $CDCl_3$

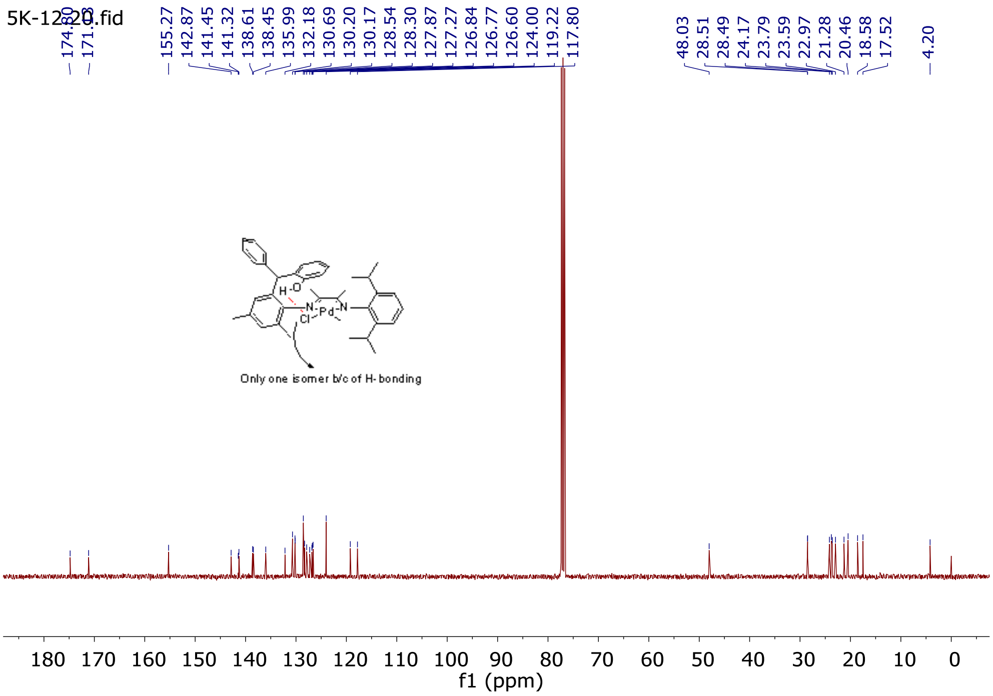

Figure S31. ^{13}C **NMR spectrum of compound Pd4 in** $CDCl_3$

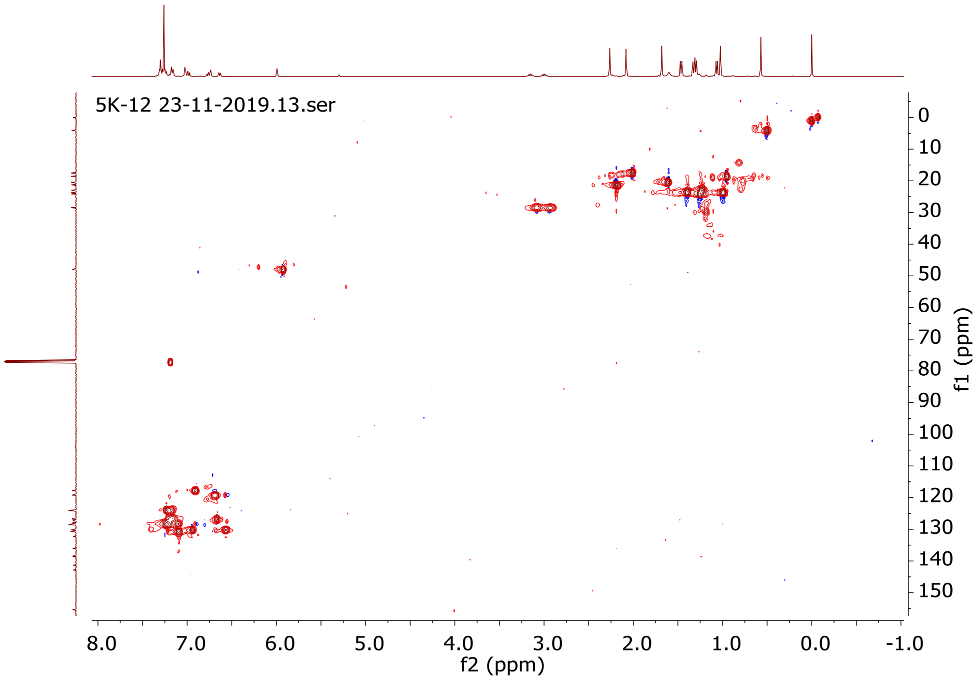

Figure S32. ^{1}H-^{13}C **HSQC NMR spectrum of compound Pd4 in** $CDCl_3$

2. MS Spectra of the Amines, Ligands and Catalysts

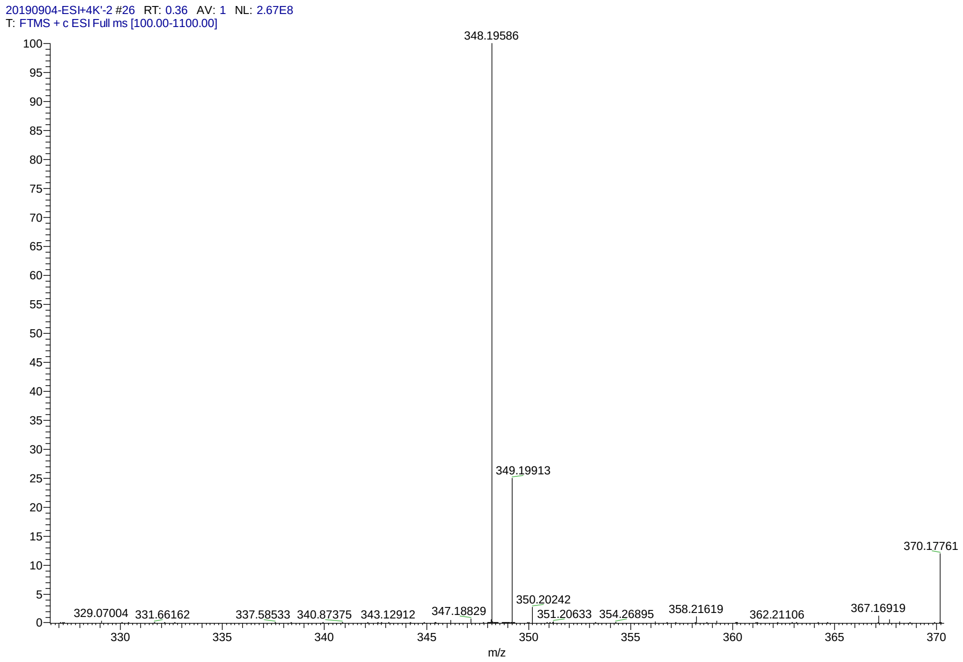

Figure S33. ESI-MS of Compound 2.

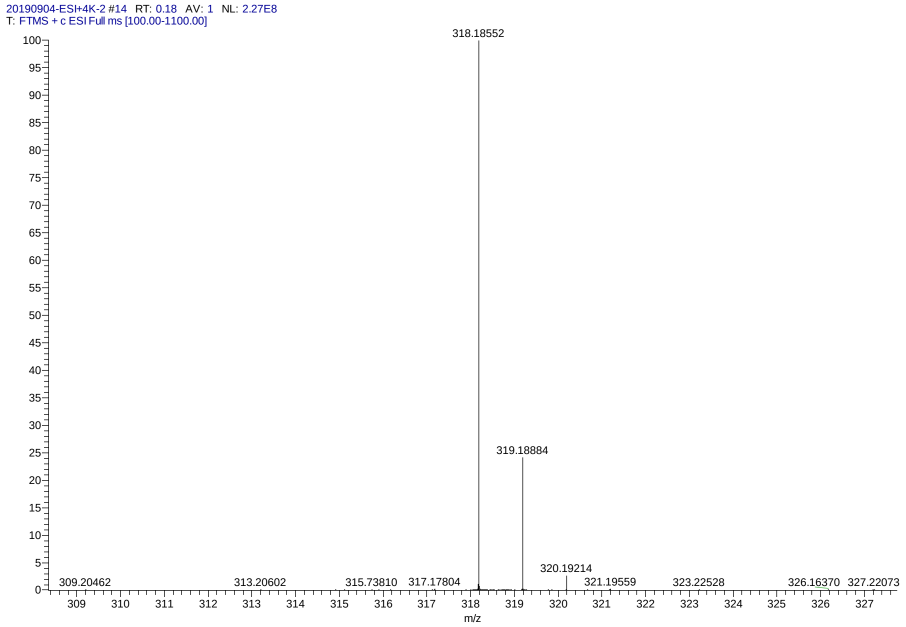

Figure S34. ESI-MS of Compound 3.

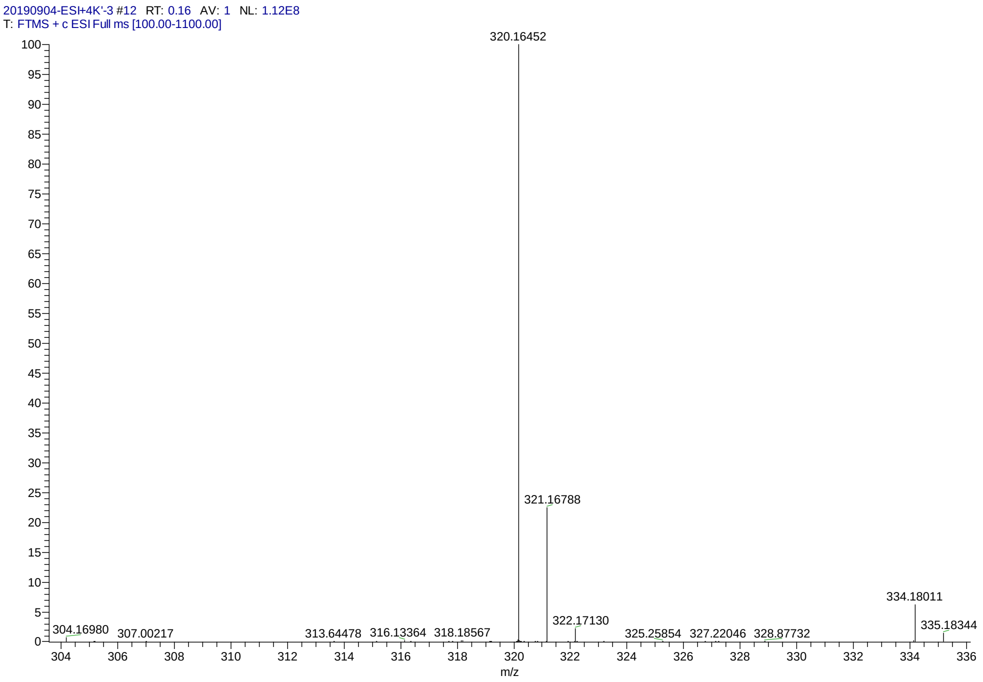

Figure S35. ESI-MS of Compound 4.

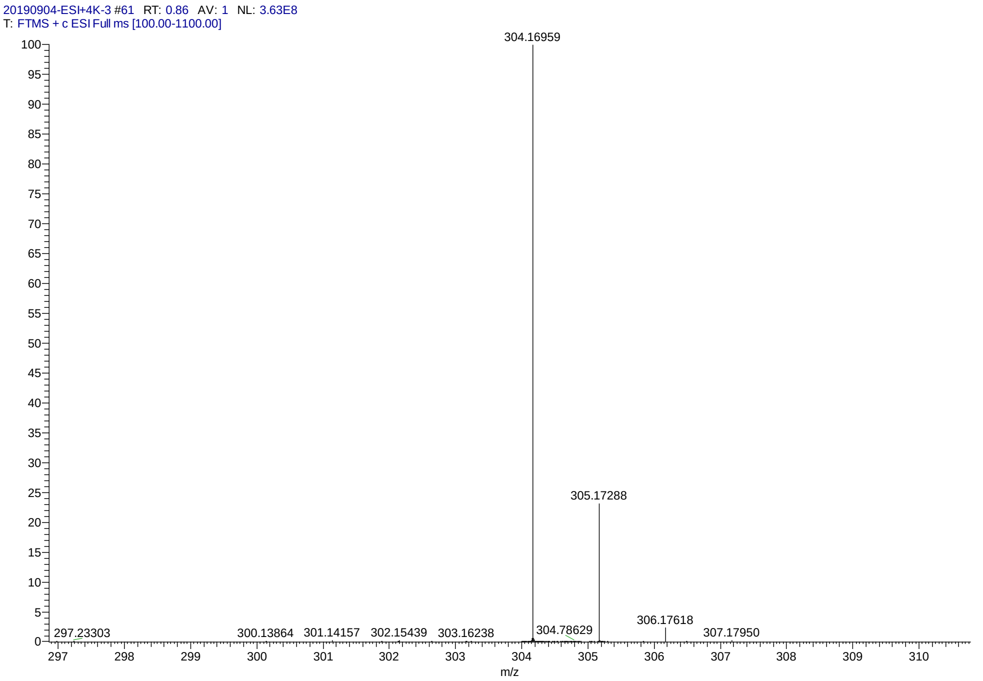

Figure S36. ESI-MS of Compound 5.

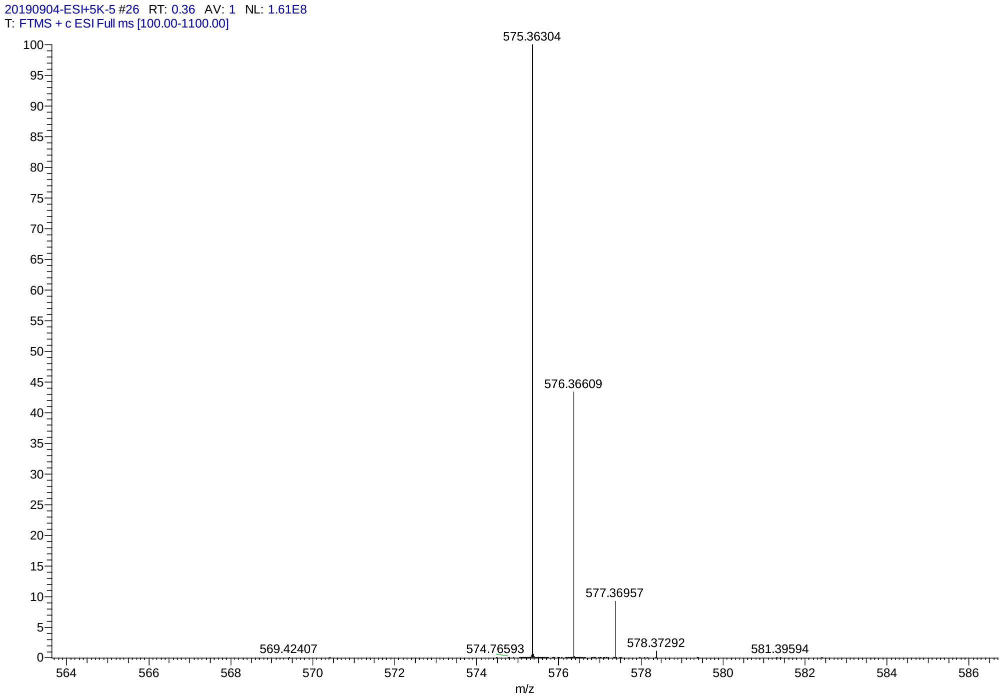

Figure S37. ESI-MS of Compound L1.

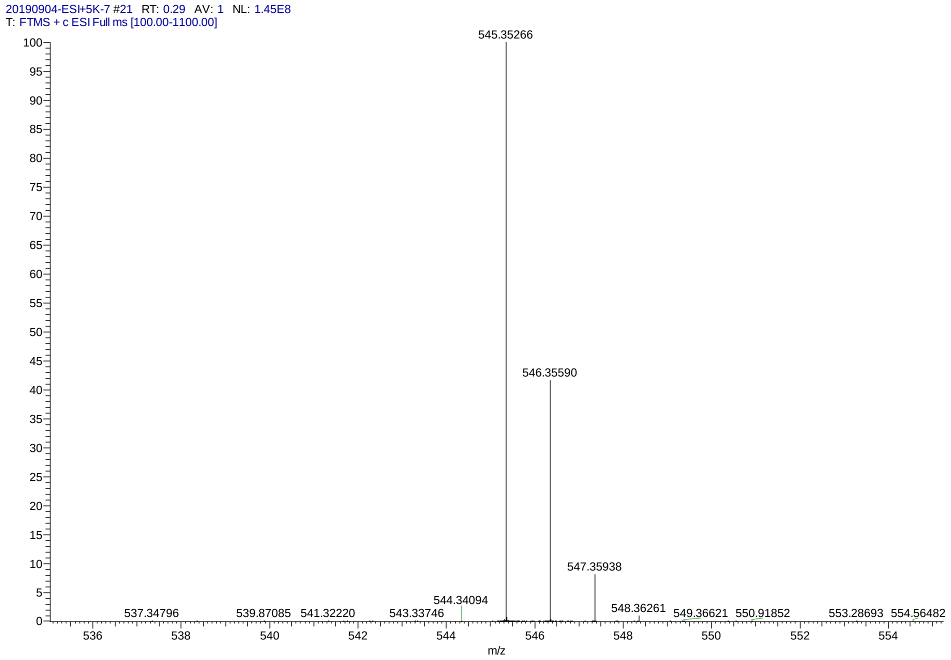

Figure S38. ESI-MS of Compound L2.

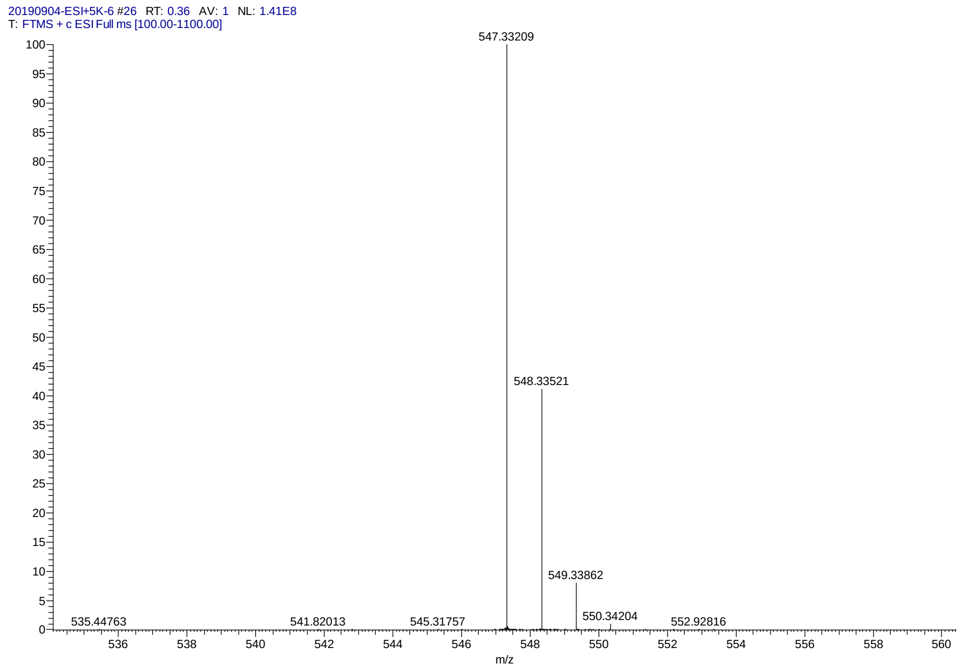

Figure S39. ESI-MS of Compound L3.

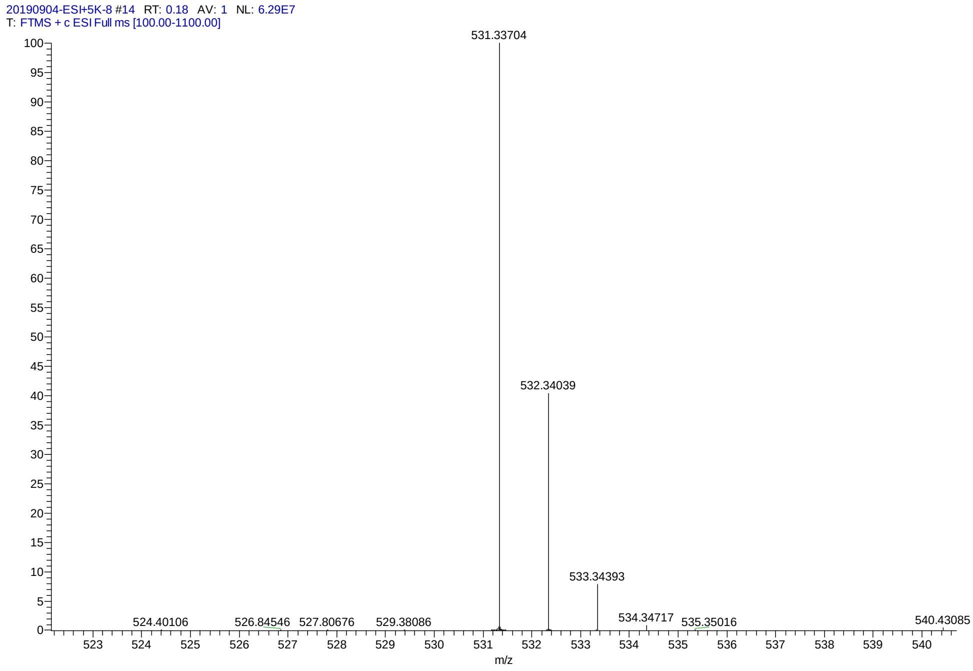

Figure S40. ESI-MS of Compound L4.

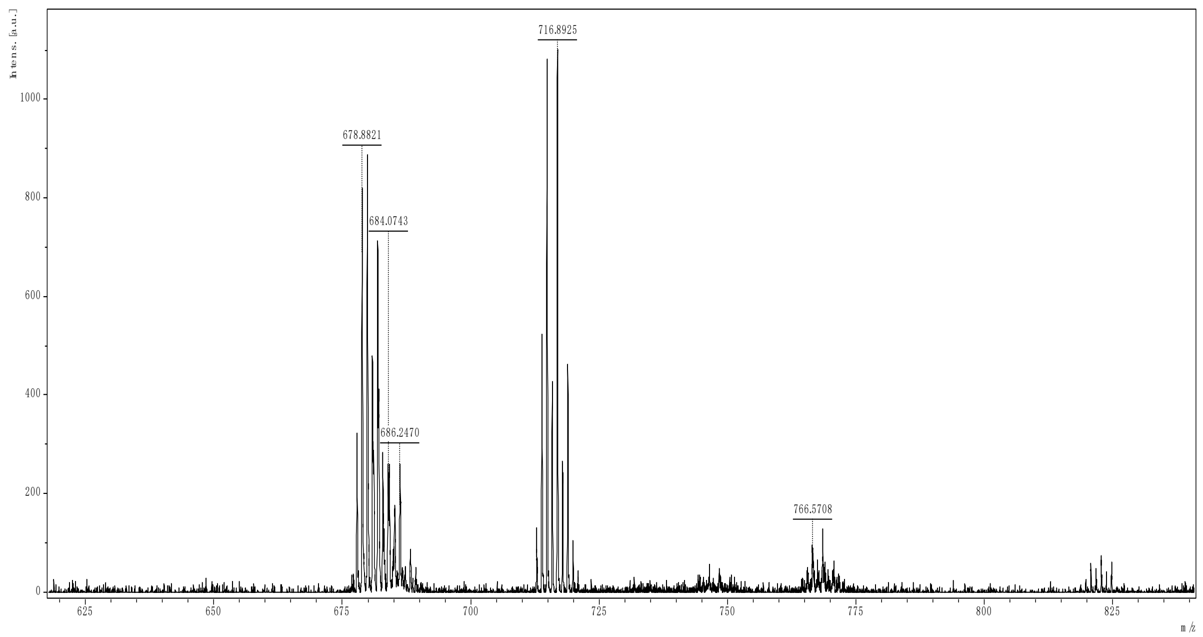

Figure S41. MALDI-TOF-MS of Pd1 Catalyst.

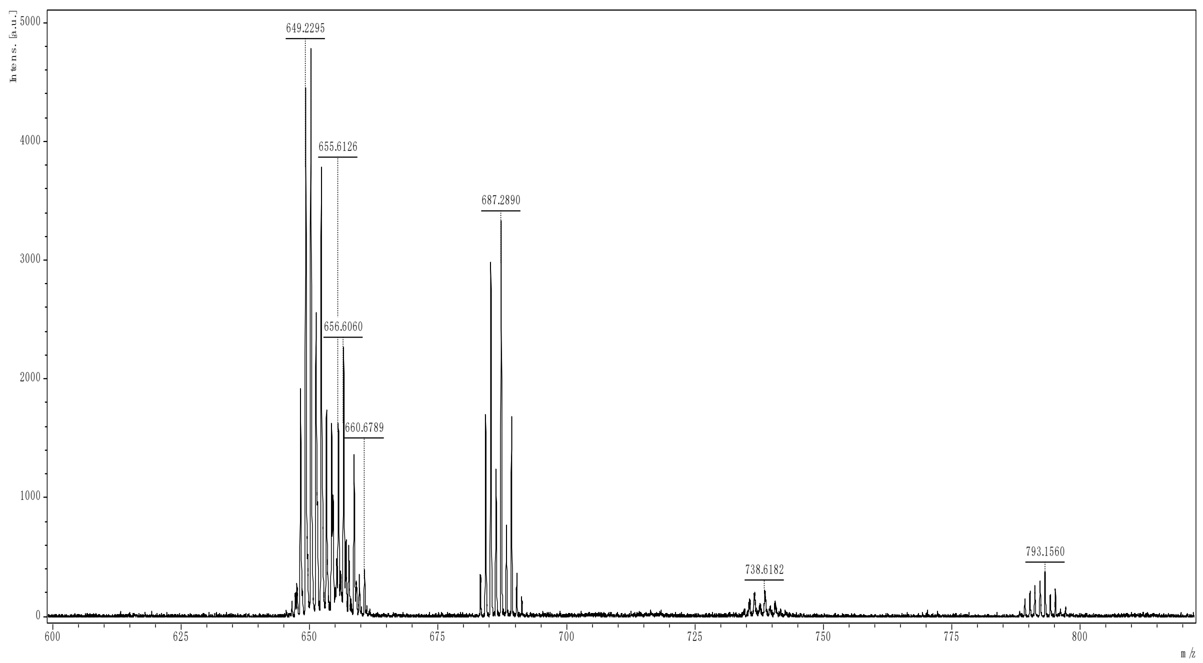

Figure S42. MALDI-TOF-MS of Pd2 Catalyst.

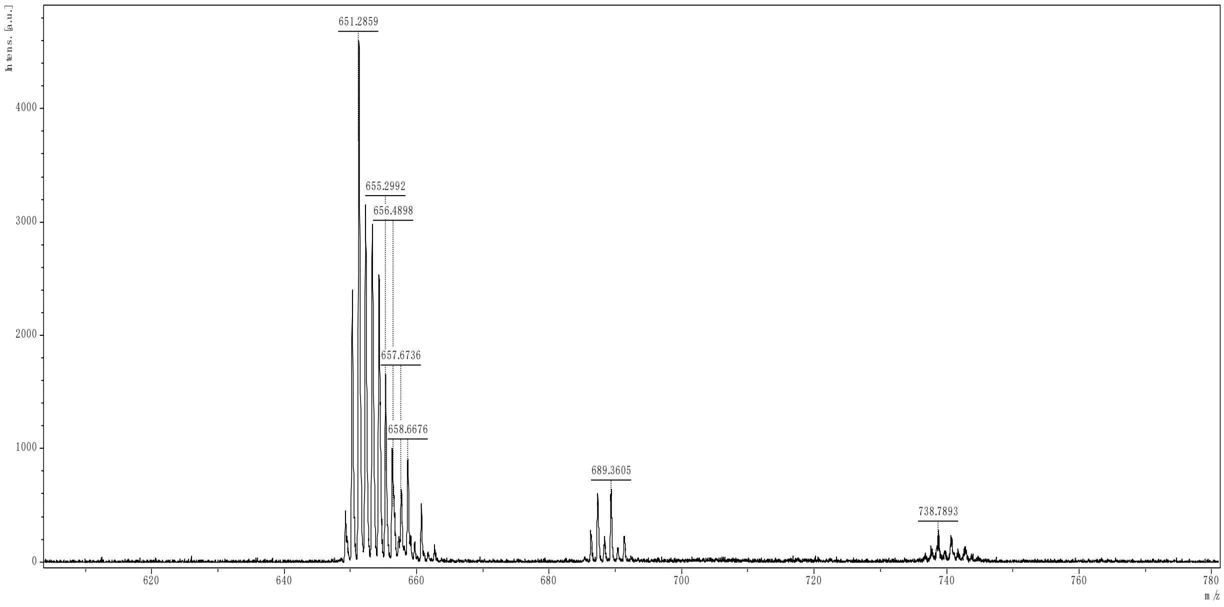

Figure S43. MALDI-TOF-MS of Pd3 Catalyst.

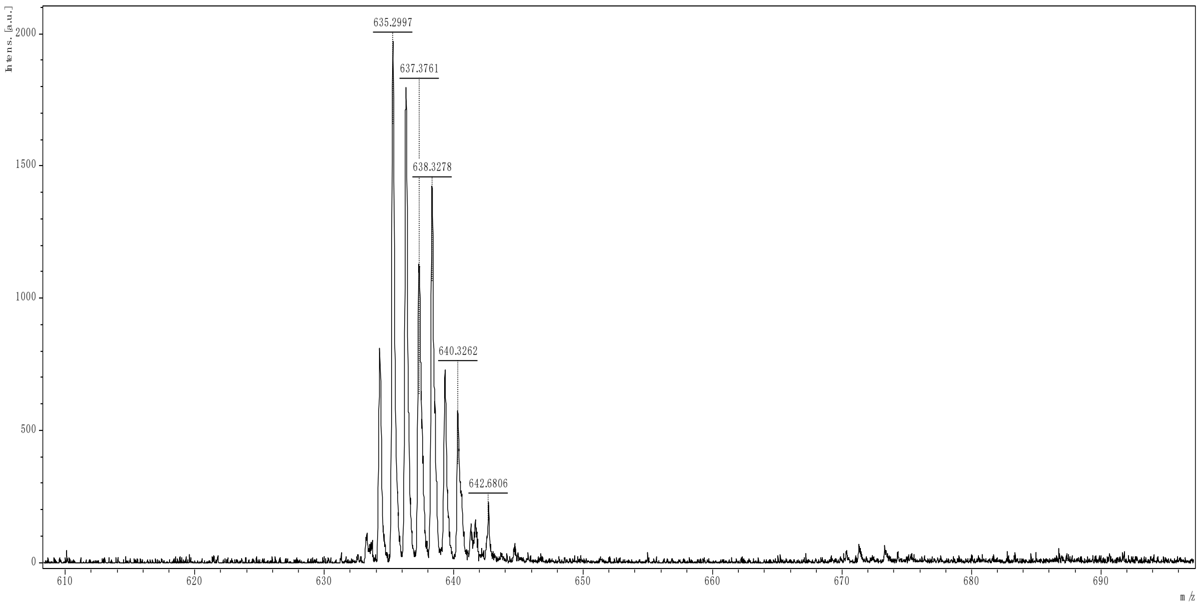

Figure S44. MALDI-TOF-MS of Pd4 Catalyst.

3. NMR Spectra of the Polymers

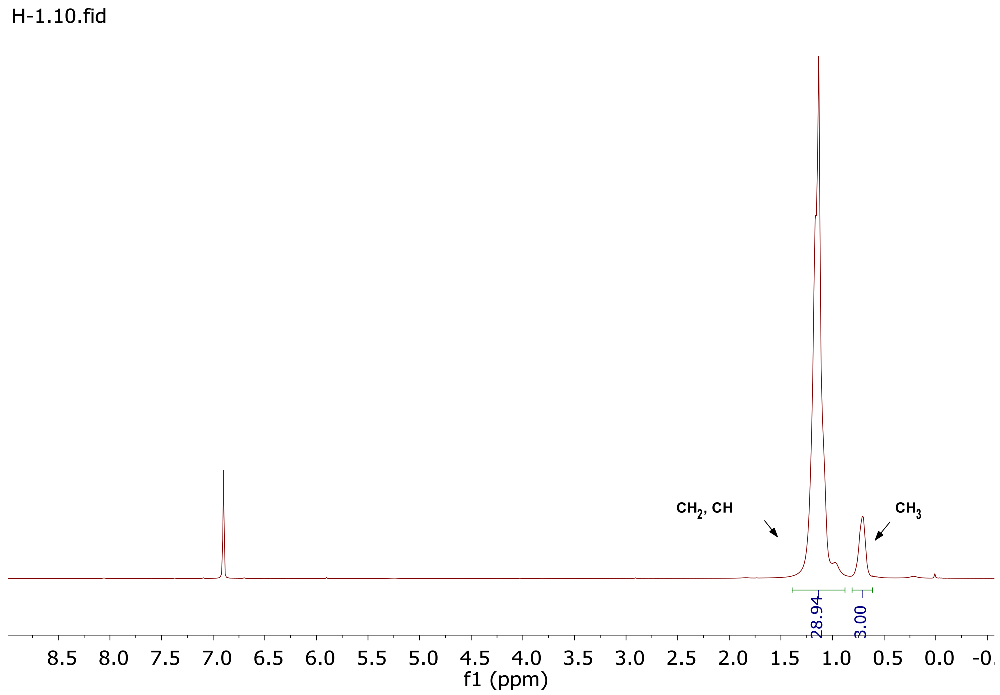

Figure S45. ^{1}H NMR spectrum of Polymer from table 1, entry 1 in C_6D_6

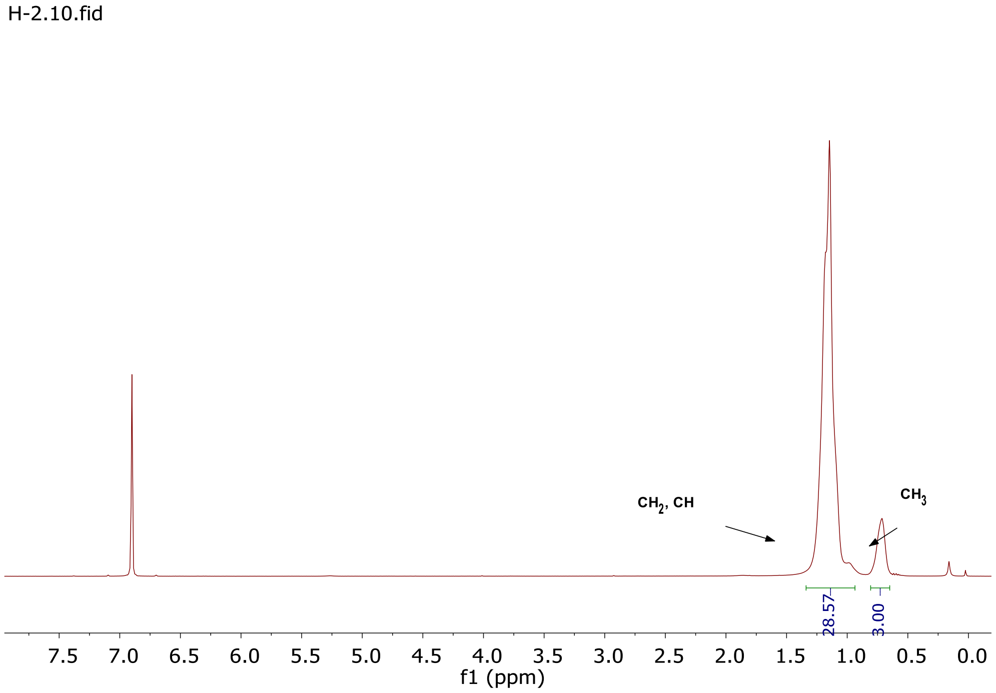

Figure S46. ^{1}H NMR spectrum of Polymer from table 1, entry 2 in C_6D_6

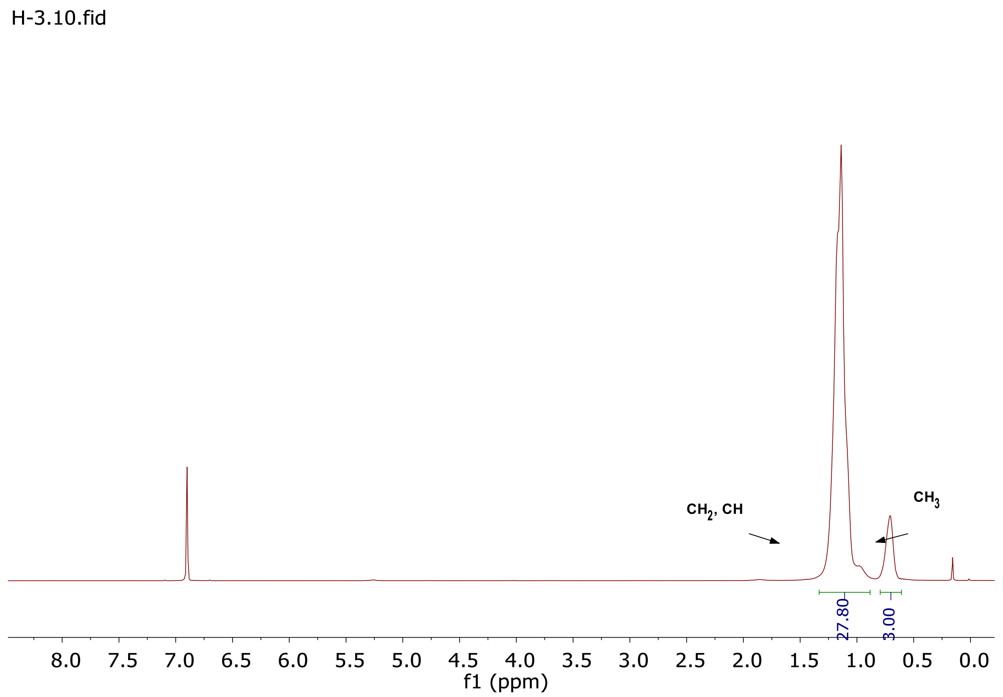

Figure S47. ^{1}**H NMR spectrum of Polymer from table 1, entry 3 in** C_6D_6

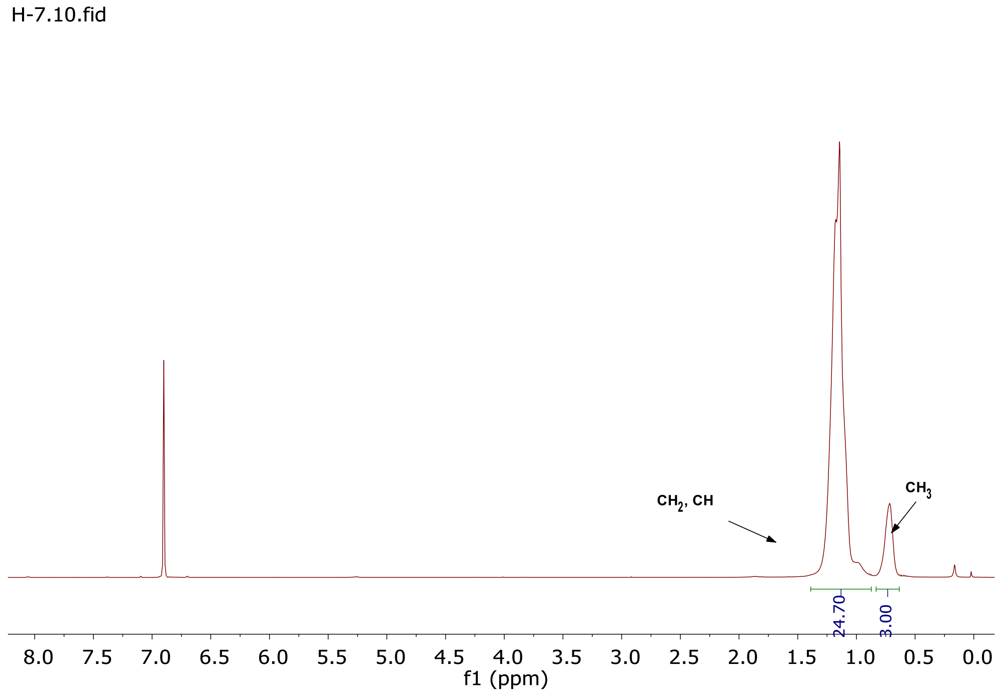

Figure S48. ^{1}**H NMR spectrum of Polymer from table 1, entry 4 in** C_6D_6

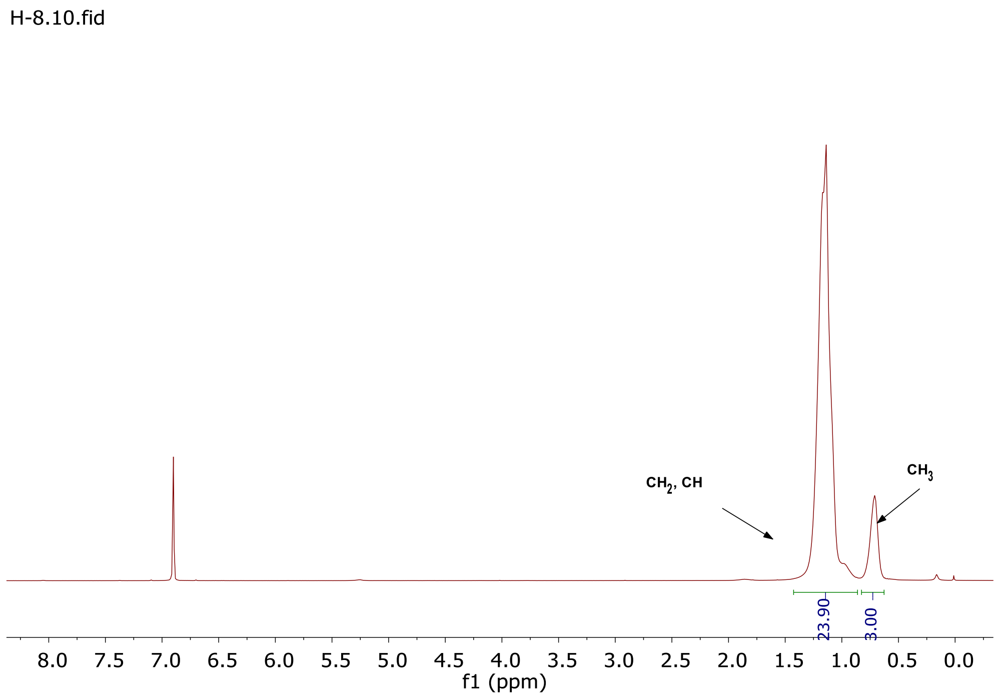

Figure S49. [1]**H NMR spectrum of Polymer from table 1, entry 5 in C_6D_6**

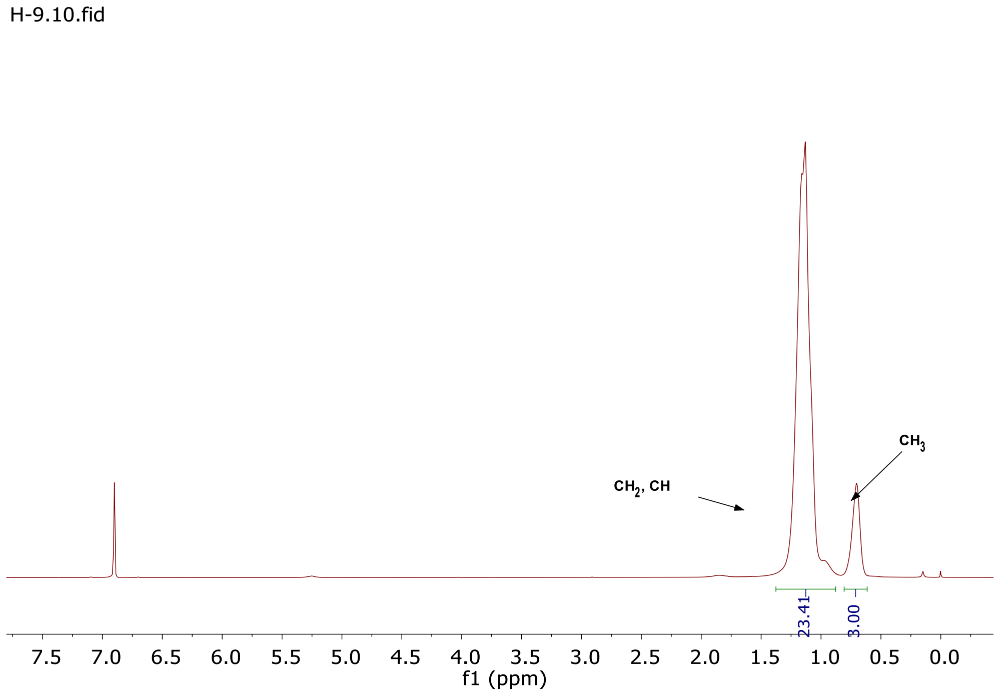

Figure S50. [1]**H NMR spectrum of Polymer from table 1, entry 6 in C_6D_6**

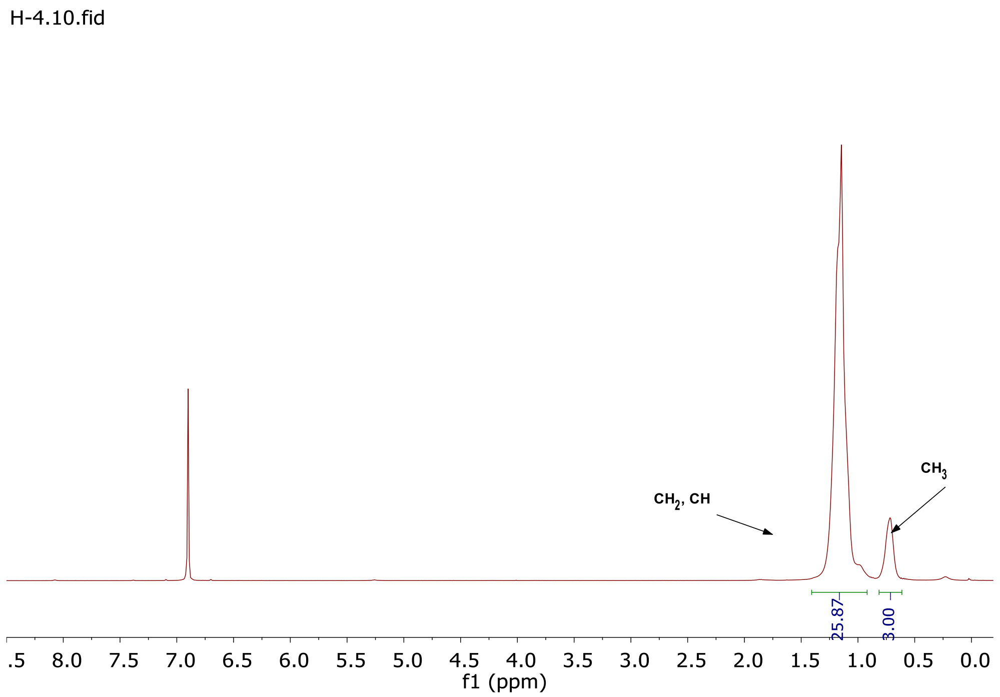

Figure S51. ^{1}**H NMR spectrum of Polymer from table 1, entry 7 in** C_6D_6

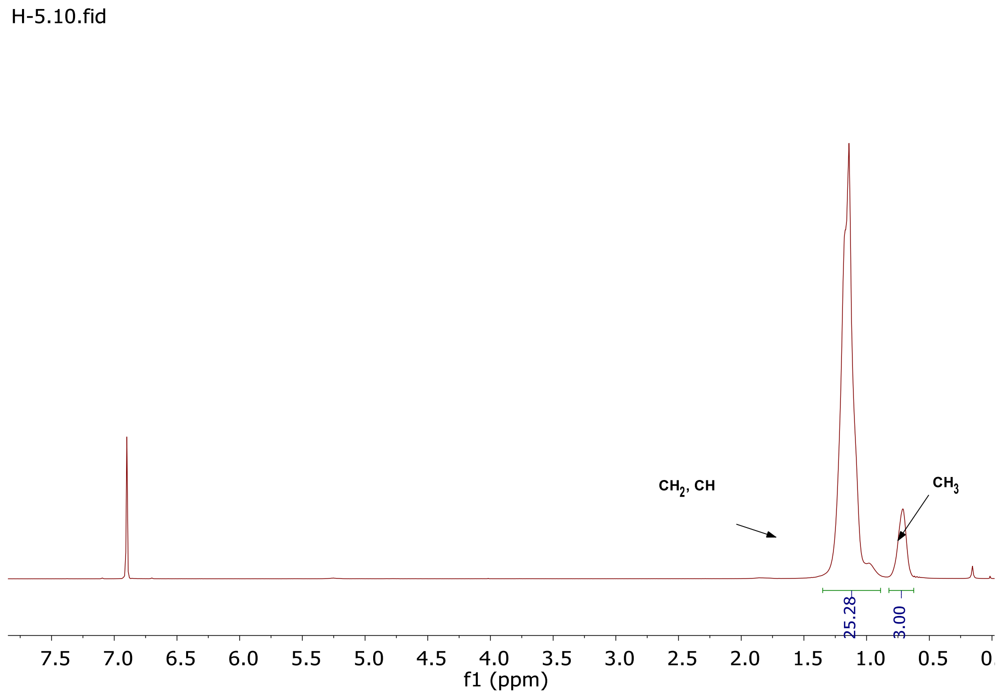

Figure S52. ^{1}**H NMR spectrum of Polymer from table 1, entry 8 in** C_6D_6

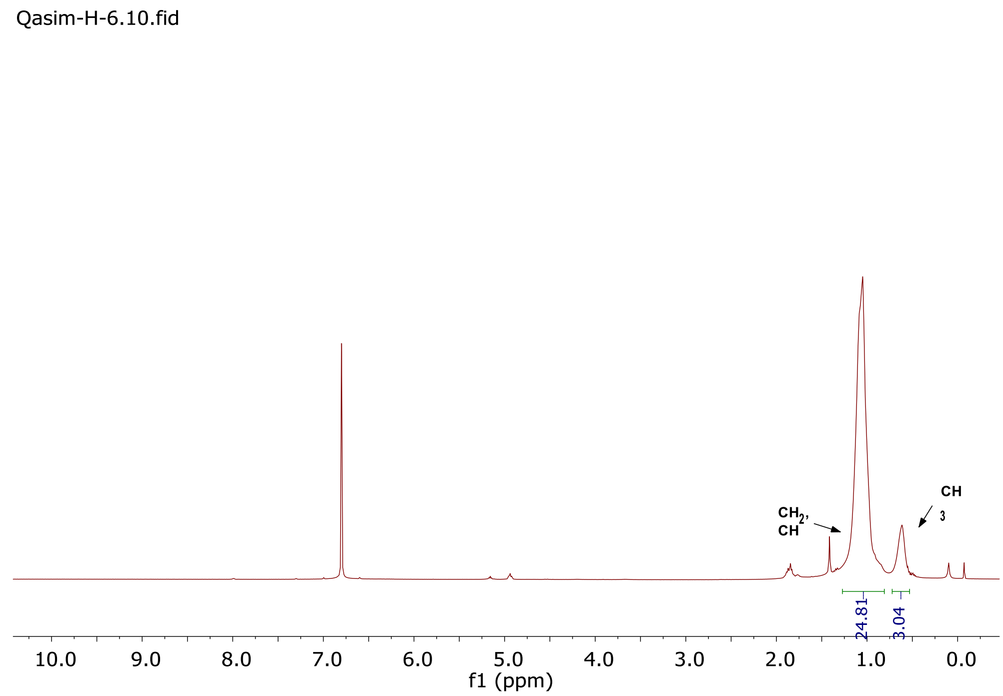

Figure S53 ¹H NMR spectrum of polymer from table 1, entry 9 in C_6D_6

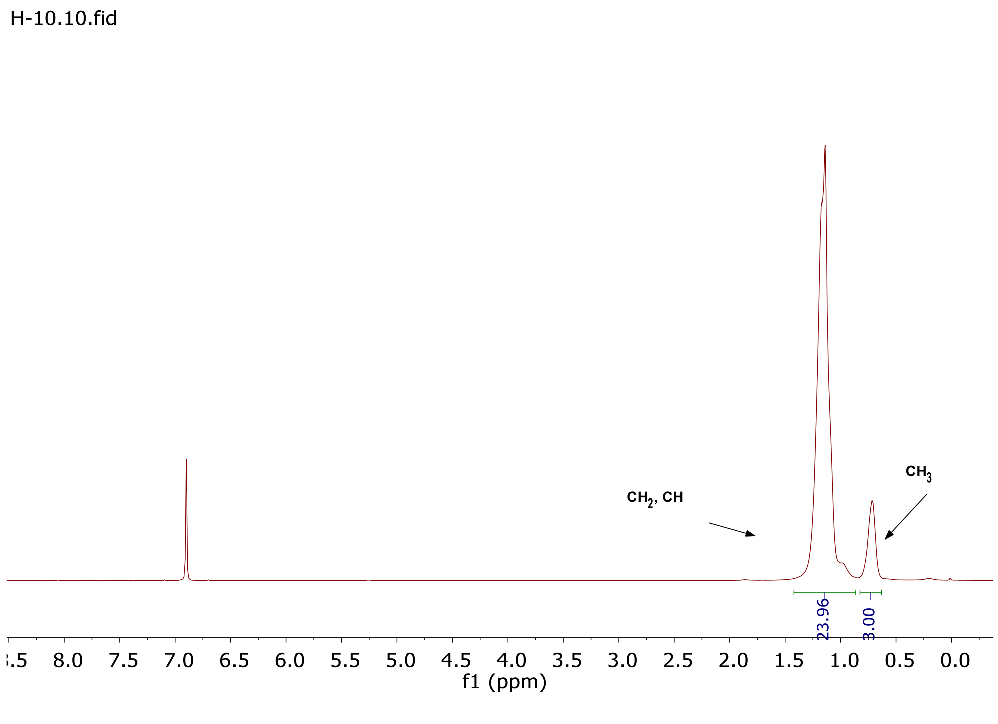

Figure S54. ¹H NMR spectrum of Polymer from table 1, entry 10 in C_6D_6

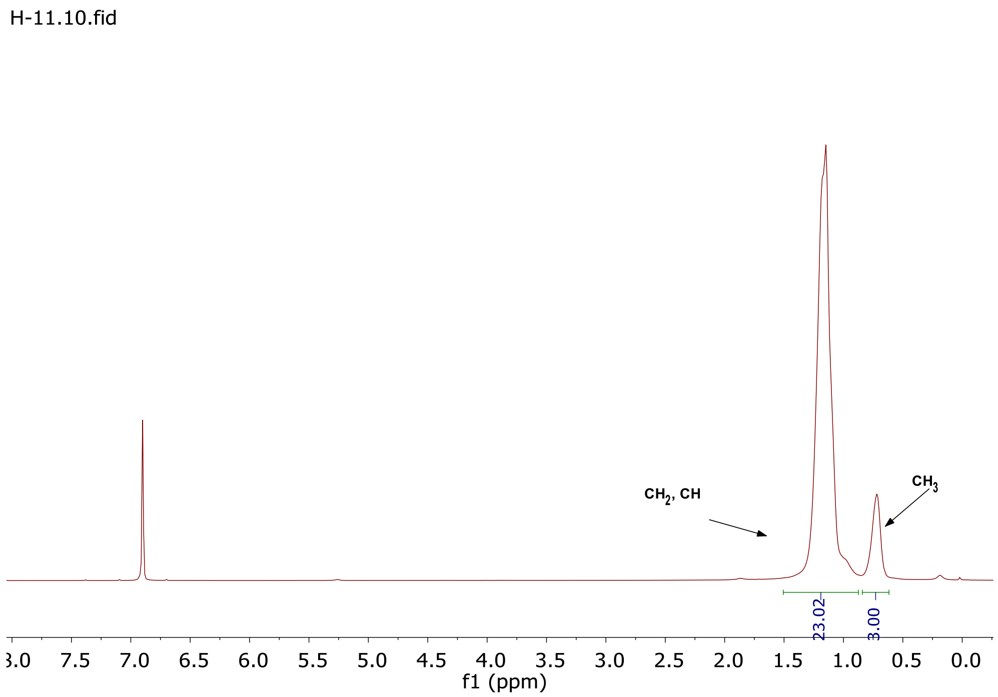

Figure S55. ^{1}H NMR spectrum of Polymer from table 1, entry 11 in C_6D_6

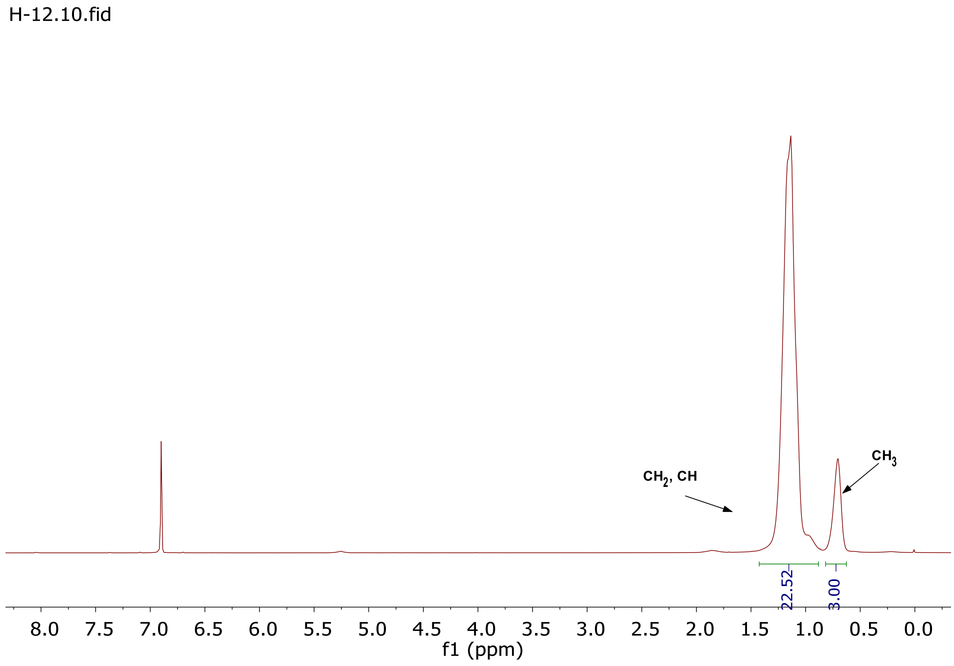

Figure S56. ^{1}H NMR spectrum of Polymer from table 1, entry 12 in C_6D_6

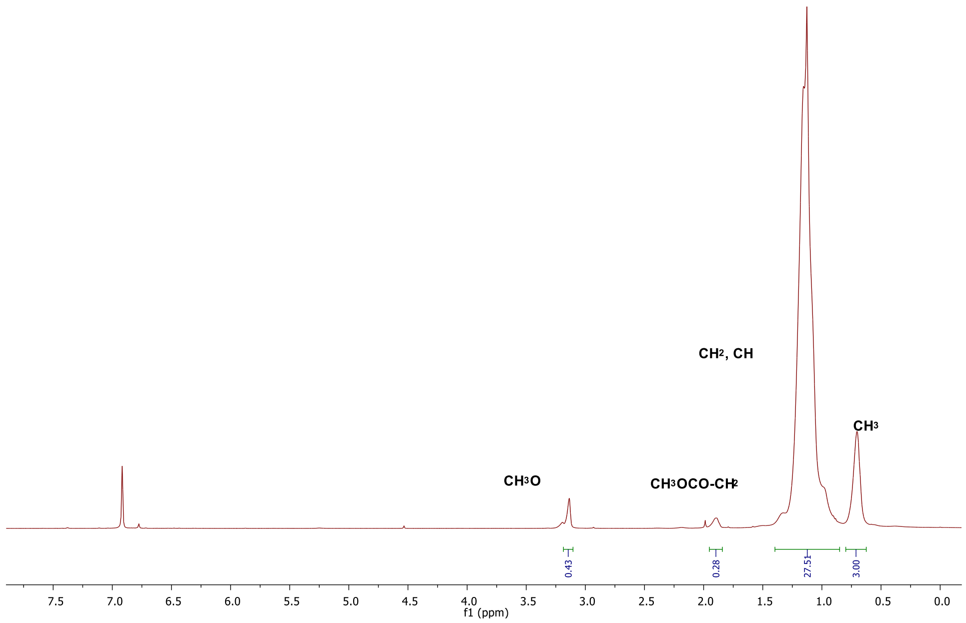

Figure S57. ^{1}**H NMR spectrum of Polymer from table 2, entry 1 in** C_6D_6

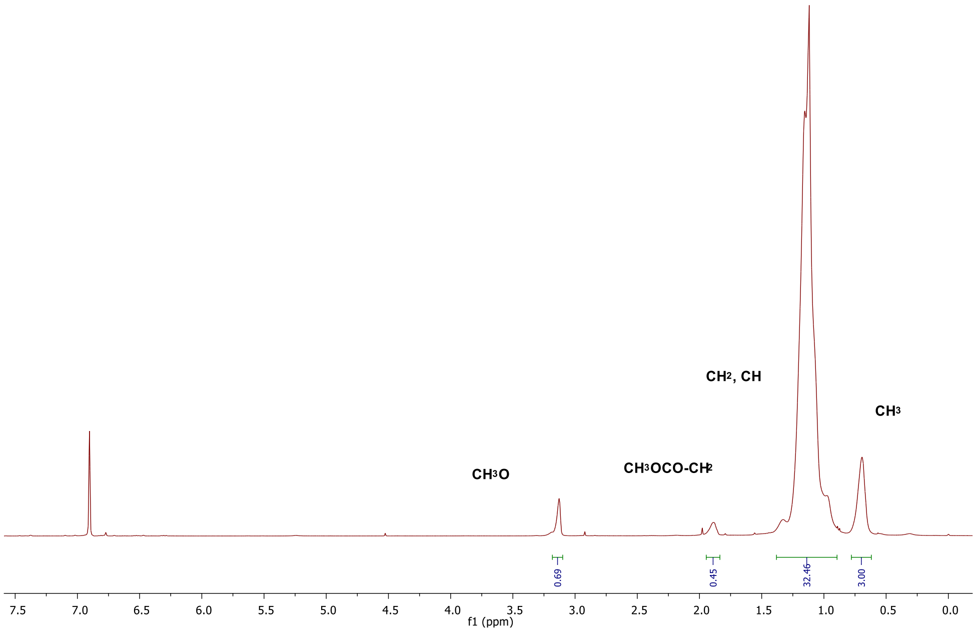

Figure S58. ^{1}**H NMR spectrum of Polymer from table 2, entry 2 in** C_6D_6

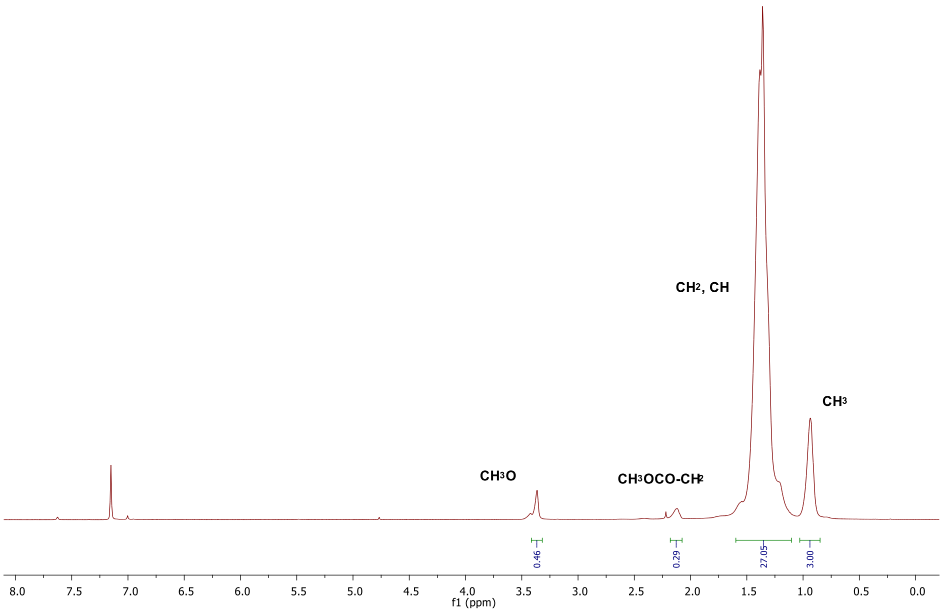

Figure S59. ^{1}**H NMR spectrum of Polymer from table 2, entry 3 in** C_6D_6

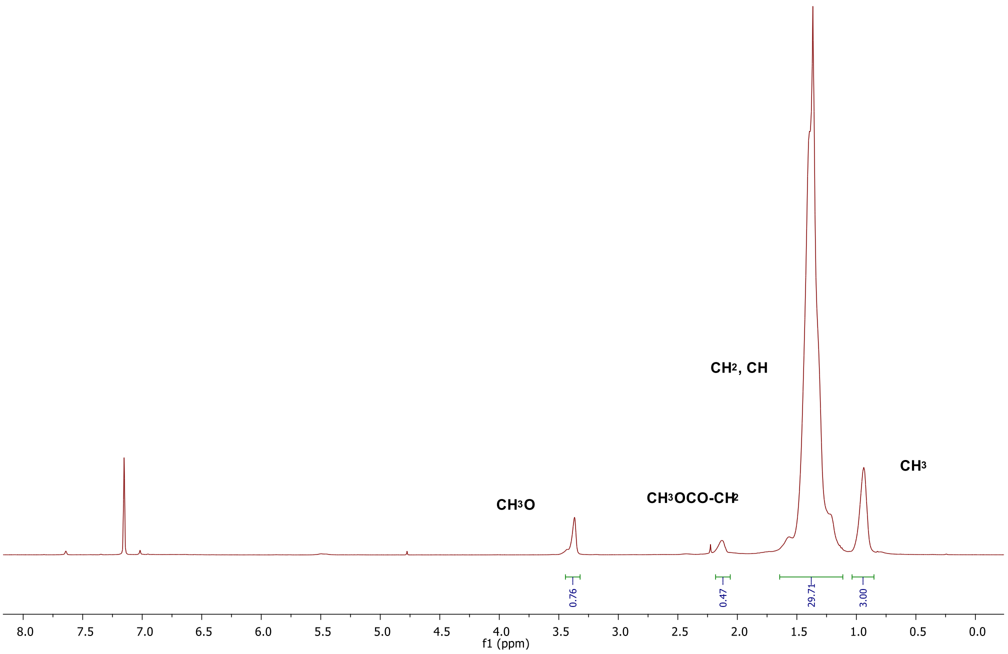

Figure S60. ^{1}**H NMR spectrum of Polymer from table 2, entry 4 in** C_6D_6

4. GPC Results of the Polymers

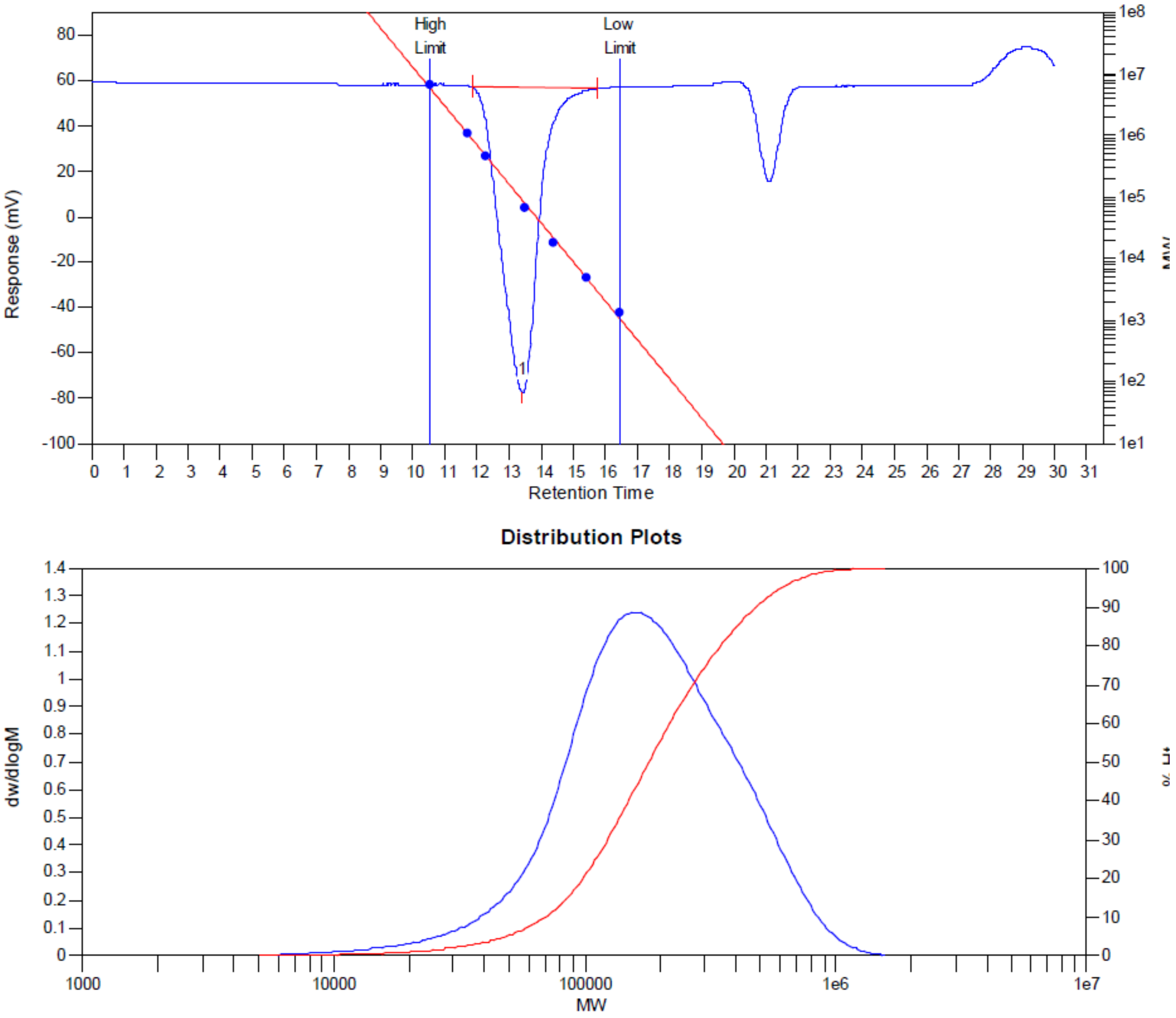

MW Averages

Peak No	Mp	Mn	Mw	Mz	Mz+1	Mv	PD
1	158759	125737	233229	374349	533412	215038	1.8549

Processed Peaks

Peak No	Name	Start RT (mins)	Max RT (mins)	End RT (mins)	Pk Height (mV)	% Height	Area (mV.secs)	% Area
1		11.85	13.42	15.77	-134.037	0	10186.3	100

Figure S61. GPC of polymer from table 1, entry 1

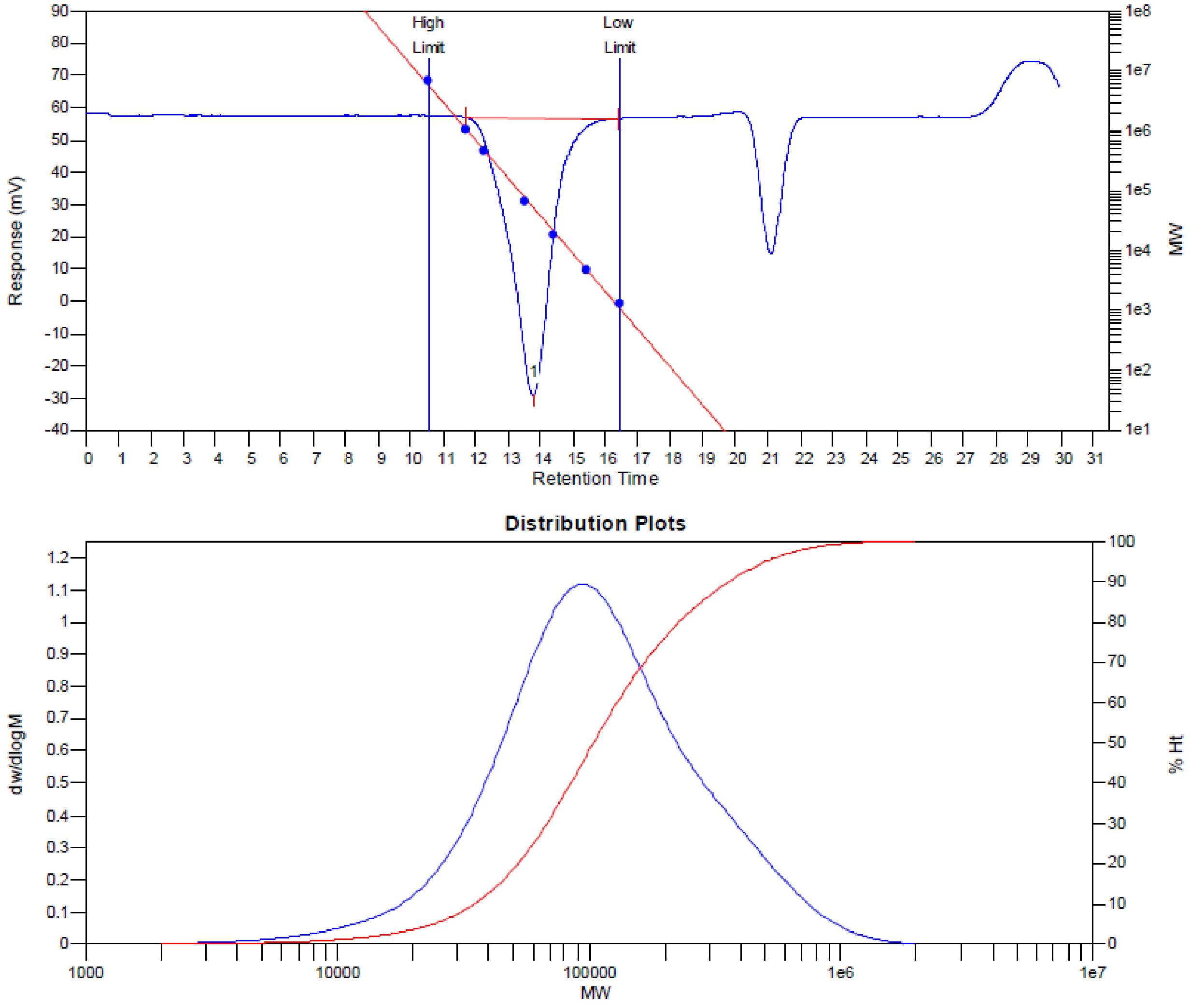

MW Averages

Peak No	Mp	Mn	Mw	Mz	Mz+1	Mv	PD
1	92781	67480	158970	337936	583175	140210	2.35581

Processed Peaks

Peak No	Name	Start RT (mins)	Max RT (mins)	End RT (mins)	Pk Height (mV)	% Height	Area (mV.secs)	% Area
1		11.70	13.78	16.40	-86.1149	0	7266.43	100

Figure S62. GPC of polymer from table 1, entry 2

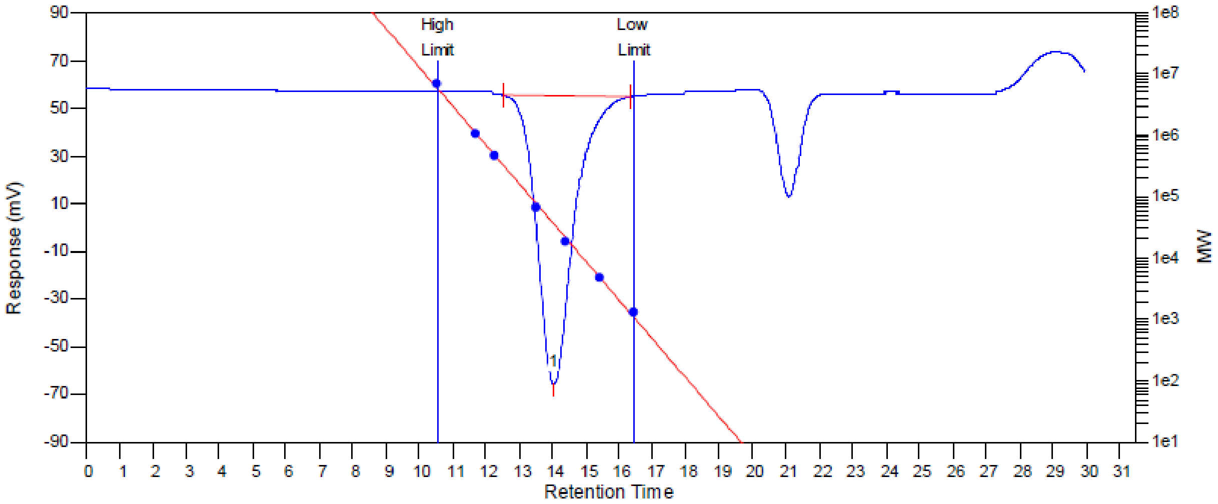

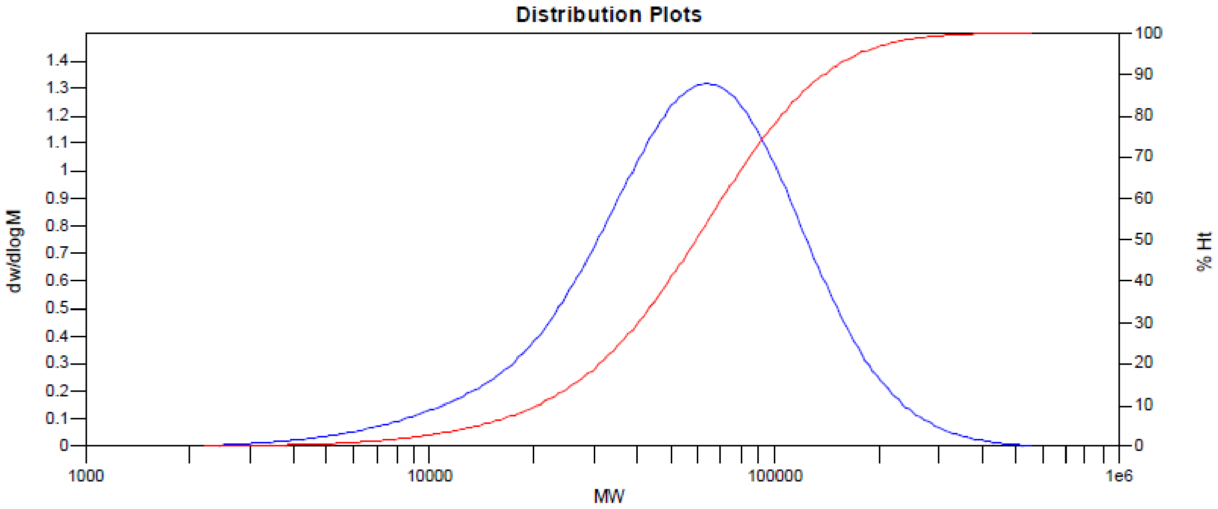

MW Averages

Peak No	Mp	Mn	Mw	Mz	Mz+1	Mv	PD
1	62778	39104	71584	112136	162796	66318	1.83061

Processed Peaks

Peak No	Name	Start RT (mins)	Max RT (mins)	End RT (mins)	Pk Height (mV)	% Height	Area (mV.secs)	% Area
1		12.57	14.05	16.33	-121.197	0	8680.86	100

Figure S63. GPC of polymer from table 1, entry 3

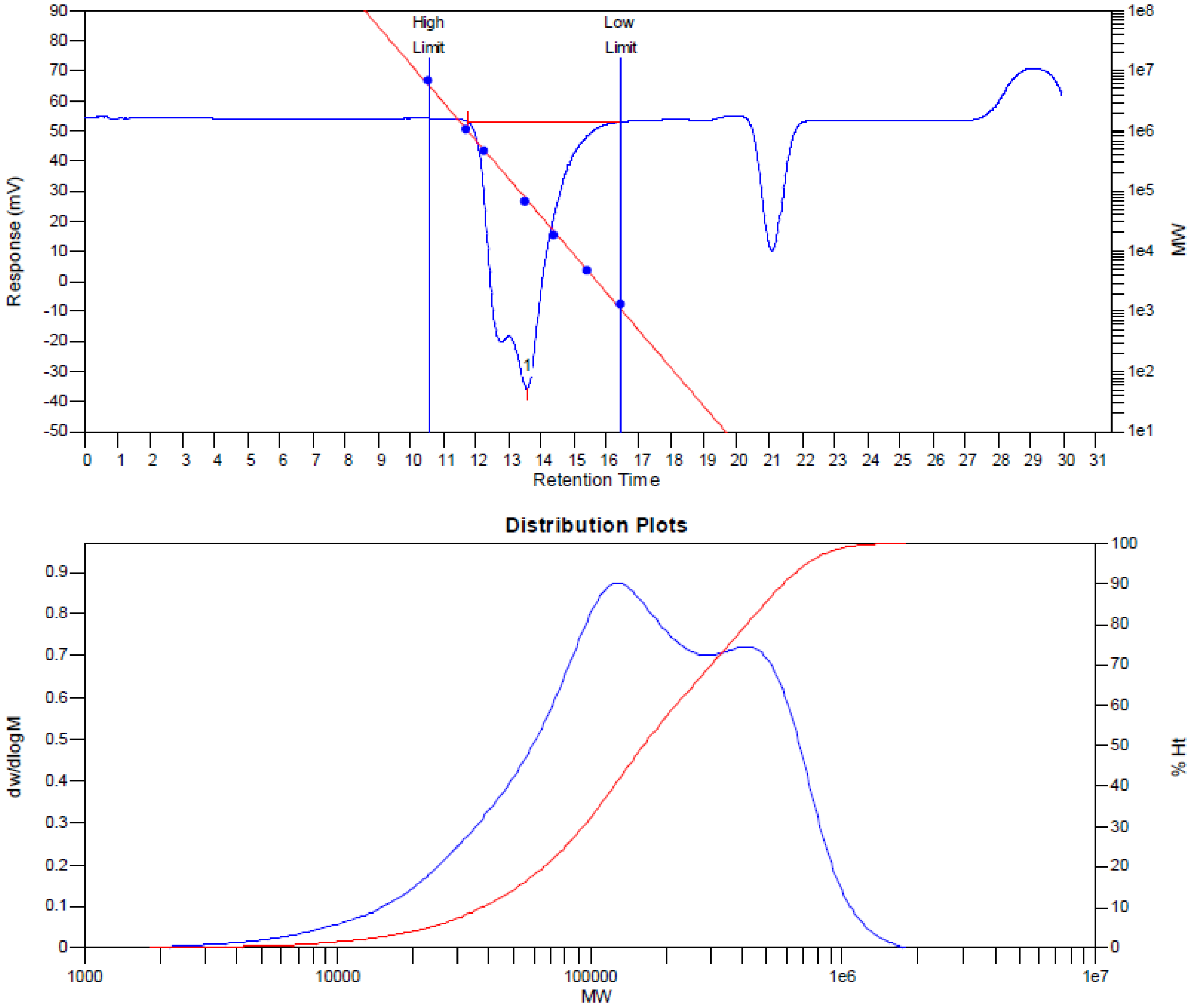

MW Averages

Peak No	Mp	Mn	Mw	Mz	Mz+1	Mv	PD
1	127440	78332	244903	461021	647634	215743	3.12647

Processed Peaks

Peak No	Name	Start RT (mins)	Max RT (mins)	End RT (mins)	Pk Height (mV)	% Height	Area (mV.secs)	% Area
1		11.77	13.57	16.47	-88.5686	0	9546.11	100

Figure S64. GPC of polymer from table 1, entry 4

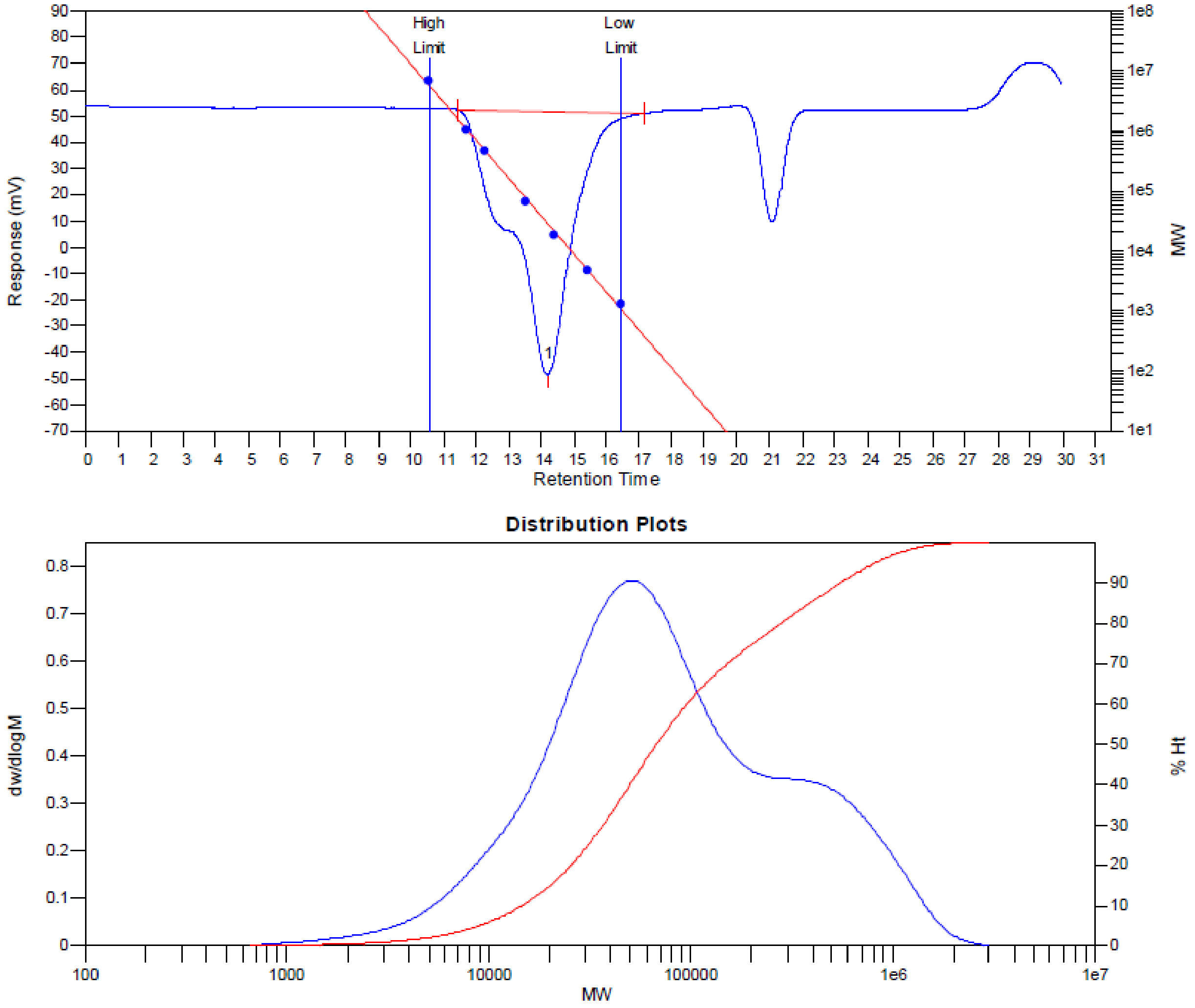

MW Averages

Peak No	Mp	Mn	Mw	Mz	Mz+1	Mv	PD
1	50393	31033	186525	631363	1033765	144324	6.01054

Processed Peaks

Peak No	Name	Start RT (mins)	Max RT (mins)	End RT (mins)	Pk Height (mV)	% Height	Area (mV.secs)	% Area
1		11.42	14.20	17.17	-100.536	0	12296.1	100

Figure S65. GPC of polymer from table 1, entry 5

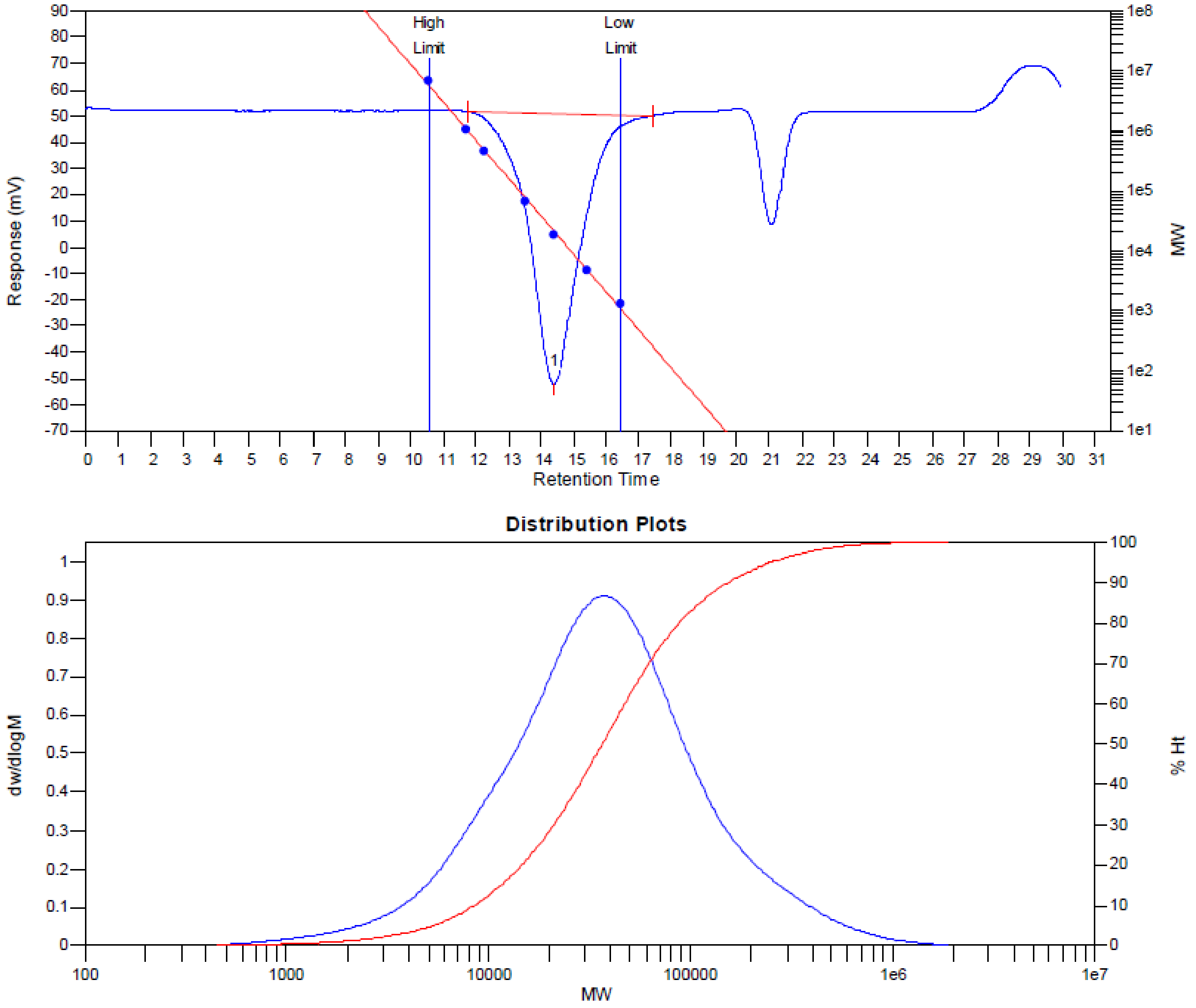

MW Averages

Peak No	Mp	Mn	Mw	Mz	Mz+1	Mv	PD
1	37595	17604	68548	240611	554935	55832	3.89389

Processed Peaks

Peak No	Name	Start RT (mins)	Max RT (mins)	End RT (mins)	Pk Height (mV)	% Height	Area (mV.secs)	% Area
1		11.73	14.40	17.42	-102.936	0	10667.8	100

Figure S66. GPC of polymer from table 1, entry 6

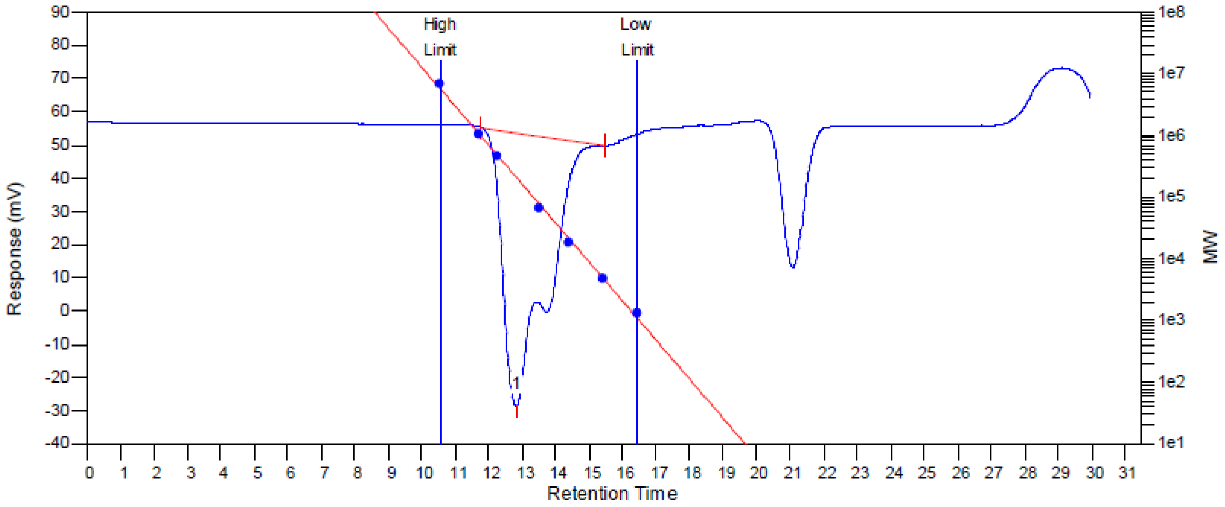

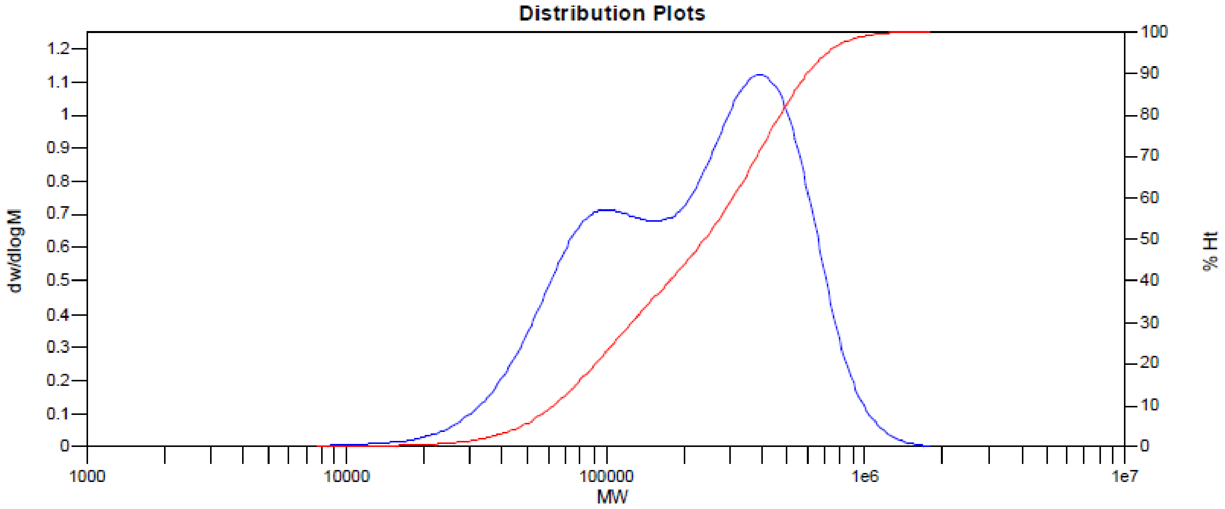

MW Averages

Peak No	Mp	Mn	Mw	Mz	Mz+1	Mv	PD
1	391803	138728	289259	458622	600572	264249	2.08508

Processed Peaks

Peak No	Name	Start RT (mins)	Max RT (mins)	End RT (mins)	Pk Height (mV)	% Height	Area (mV.secs)	% Area
1		11.77	12.80	15.48	-82.4913	0	6932.73	100

Figure S67. GPC of polymer from table 1, entry 7

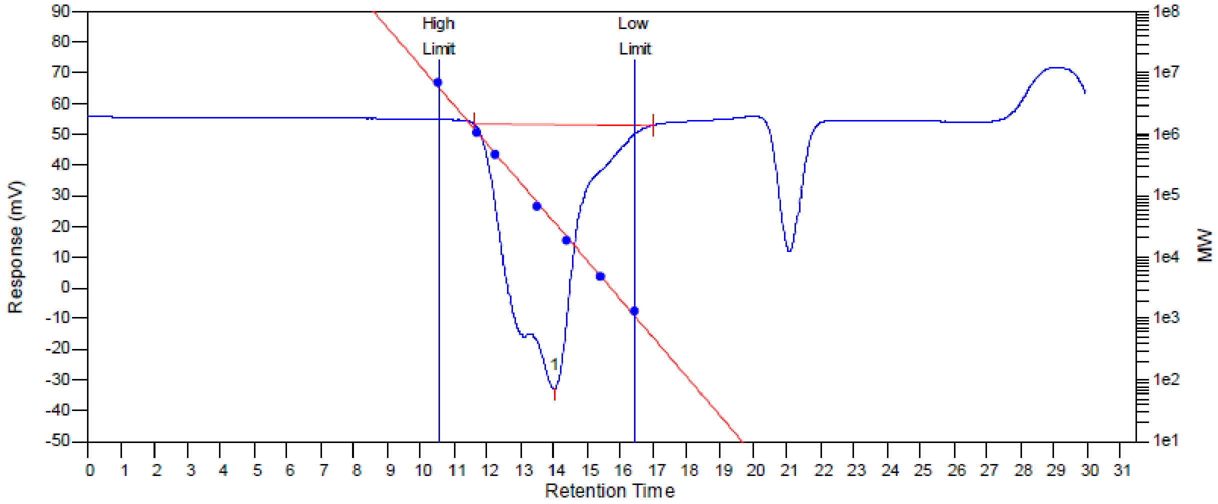

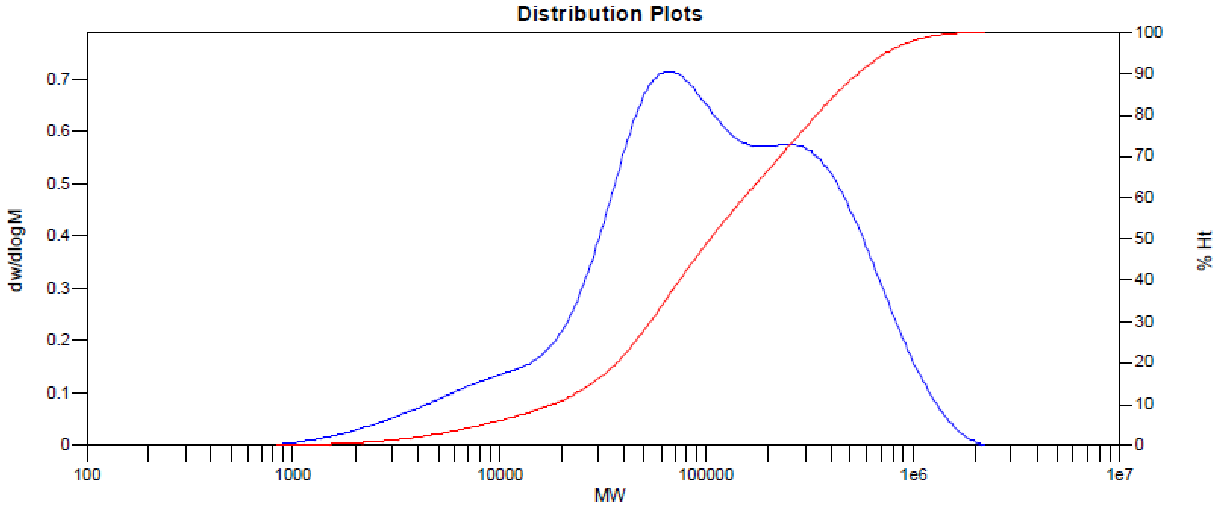

MW Averages

Peak No	Mp	Mn	Mw	Mz	Mz+1	Mv	PD
1	65919	35019	205938	519027	813585	170006	5.88075

Processed Peaks

Peak No	Name	Start RT (mins)	Max RT (mins)	End RT (mins)	Pk Height (mV)	% Height	Area (mV.secs)	% Area
1		11.62	14.02	17.00	-85.9394	0	11315.2	100

Figure S68. GPC of polymer from table 1, entry 8

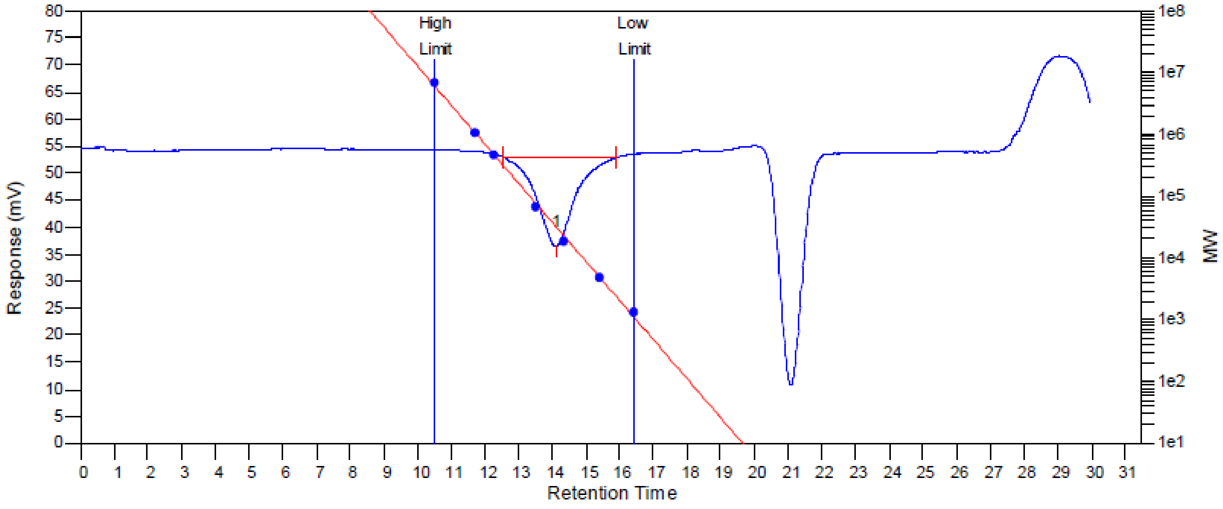

Distribution Plots

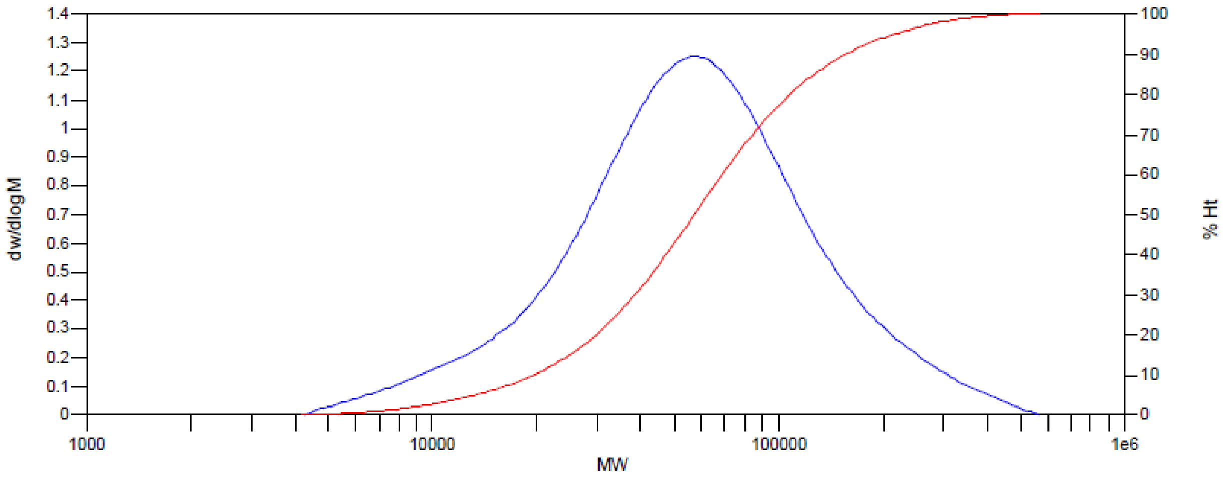

MW Averages

Peak No	Mp	Mn	Mw	Mz	Mz+1	Mv	PD
1	56936	39274	75933	135502	213075	69015	1.93342

Processed Peaks

Peak No	Name	Start RT (mins)	Max RT (mins)	End RT (mins)	Pk Height (mV)	% Height	Area (mV.secs)	% Area
1		12.55	14.12	15.90	-16.43	0	1237.2	100

Figure S69. GPC of polymer from table 1, entry 9

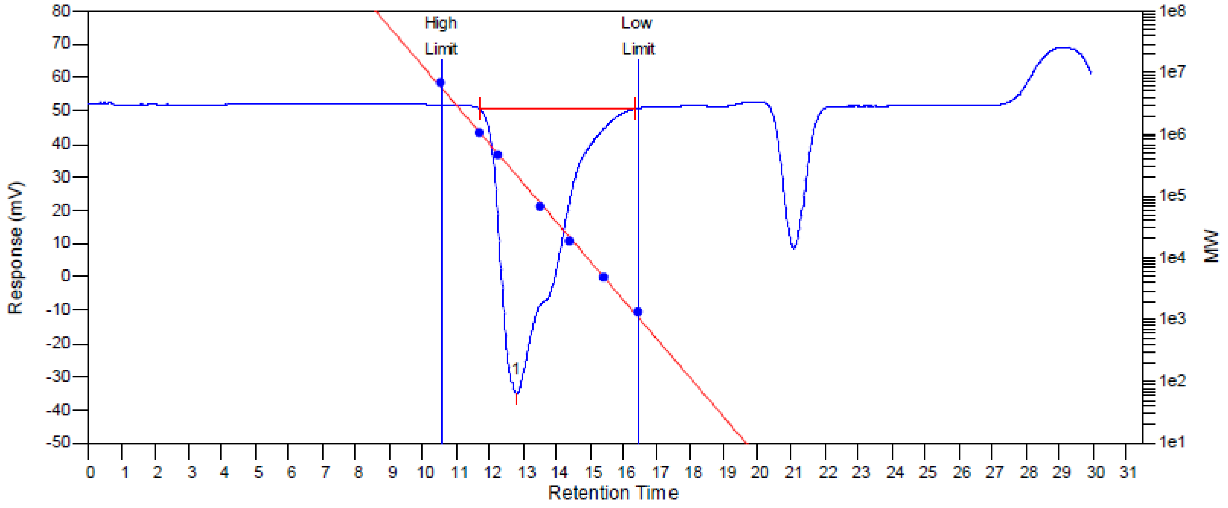

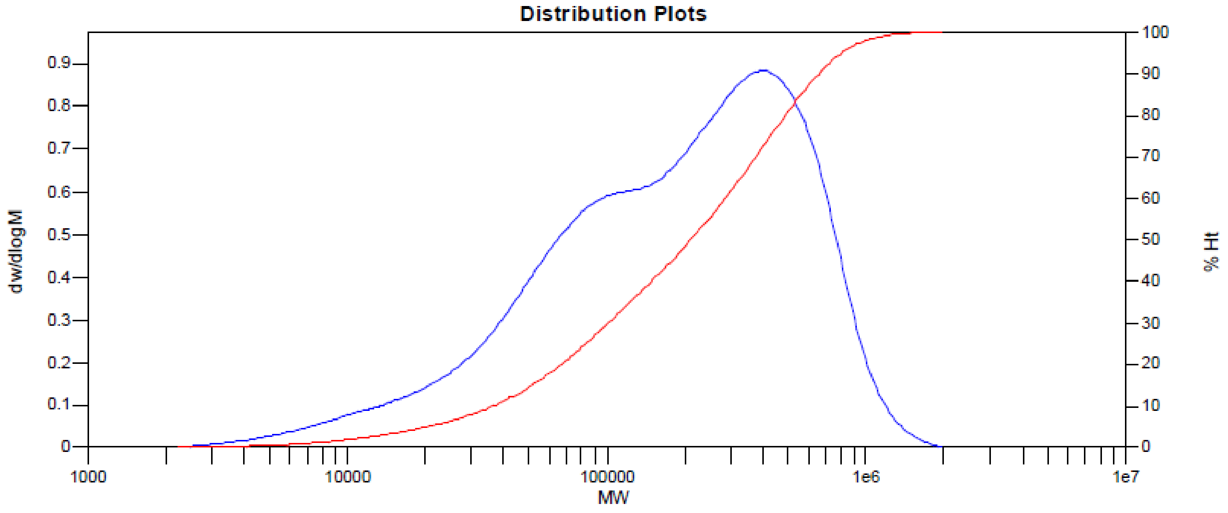

MW Averages

Peak No	Mp	Mn	Mw	Mz	Mz+1	Mv	PD
1	401487	77473	285657	519674	705561	251532	3.68718

Processed Peaks

Peak No	Name	Start RT (mins)	Max RT (mins)	End RT (mins)	Pk Height (mV)	% Height	Area (mV.secs)	% Area
1		11.70	12.78	16.33	-85.5061	0	9108.95	100

Figure S70. GPC of polymer from table 1, entry 10

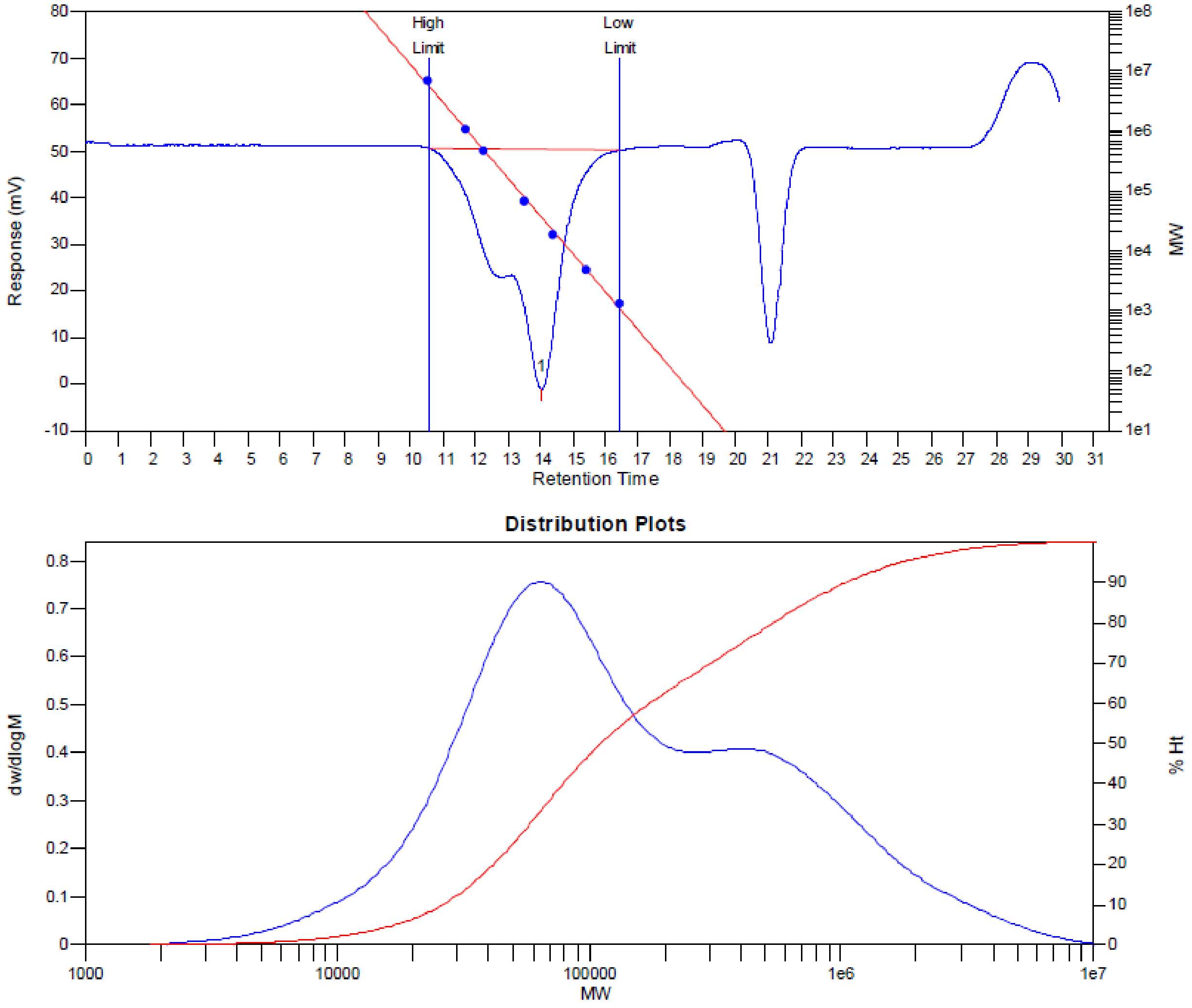

MW Averages

Peak No	Mp	Mn	Mw	Mz	Mz+1	Mv	PD
1	64329	57914	400564	1910483	3793076	291766	6.91653

Processed Peaks

Peak No	Name	Start RT (mins)	Max RT (mins)	End RT (mins)	Pk Height (mV)	% Height	Area (mV.secs)	% Area
1		10.53	14.03	16.47	-51.5148	0	6419.59	100

Figure S71. GPC of polymer from table 1, entry 11

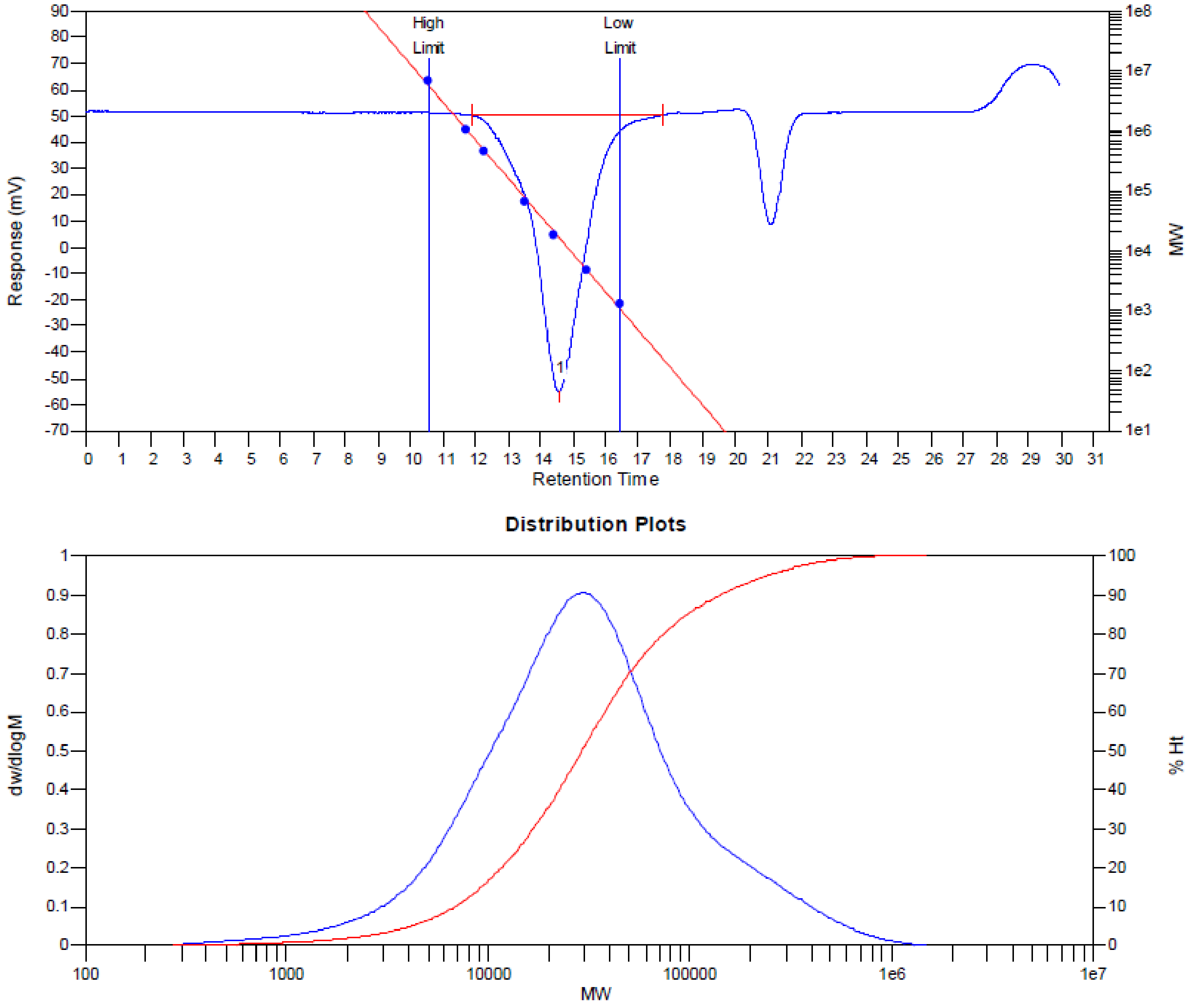

MW Averages

Peak No	Mp	Mn	Mw	Mz	Mz+1	Mv	PD
1	29450	13433	60386	224053	469034	48137	4.49535

Processed Peaks

Peak No	Name	Start RT (mins)	Max RT (mins)	End RT (mins)	Pk Height (mV)	% Height	Area (mV.secs)	% Area
1		11.90	14.57	17.77	-105.209	0	10963.9	100

Figure S72. GPC of polymer from table 1, entry 12

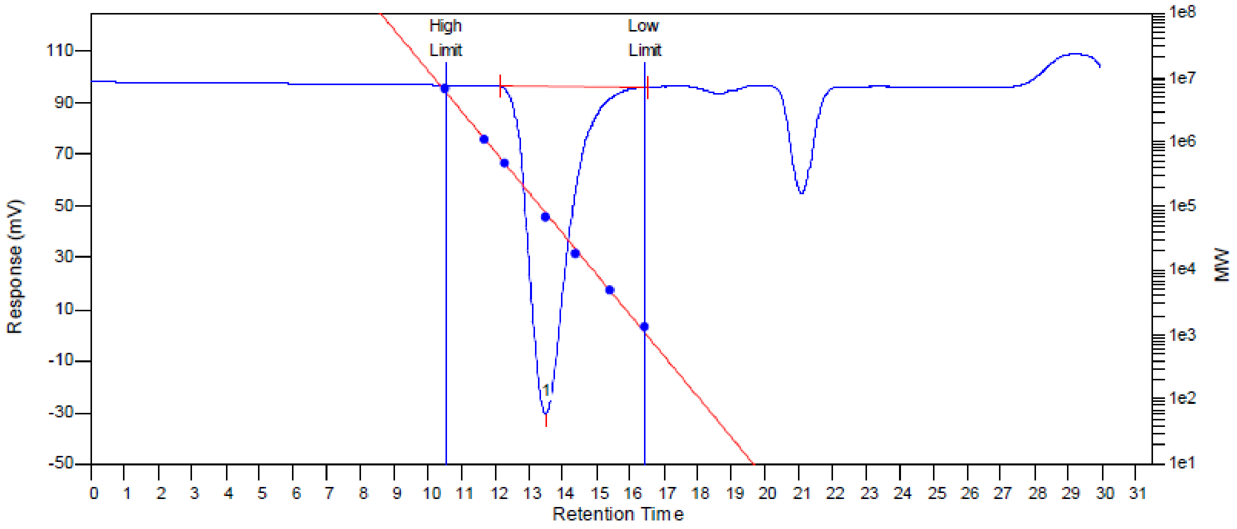

Distribution Plots

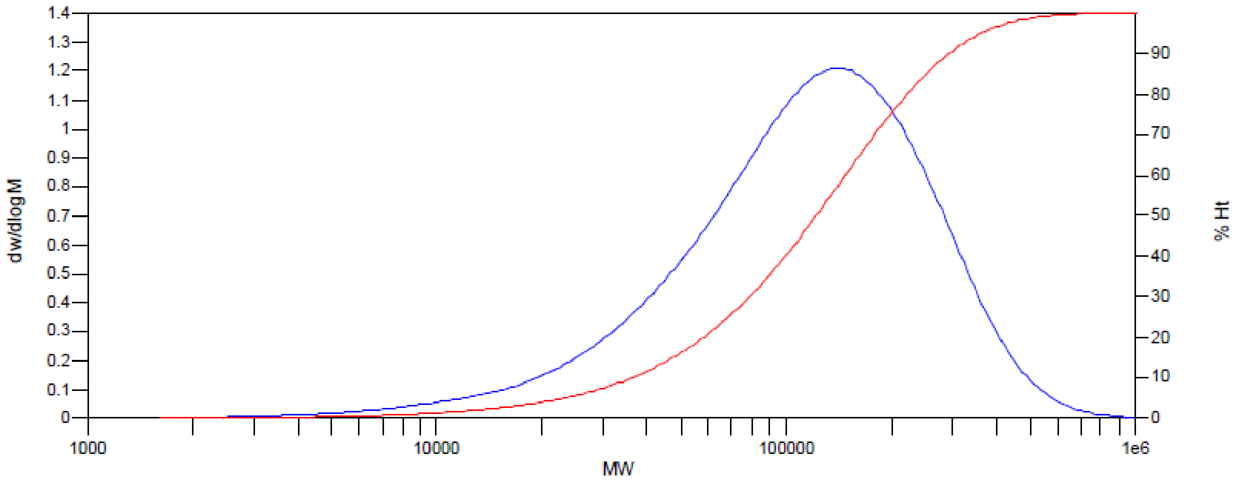

MW Averages

Peak No	Mp	Mn	Mw	Mz	Mz+1	Mv	PD
1	140514	68991	145984	226833	310388	134593	2.11599

Processed Peaks

Peak No	Name	Start RT (mins)	Max RT (mins)	End RT (mins)	Pk Height (mV)	% Height	Area (mV.secs)	% Area
1		12.15	13.50	16.55	-126.938	0	9892.06	100

Figure S73. GPC of polymer from table 2, entry 1

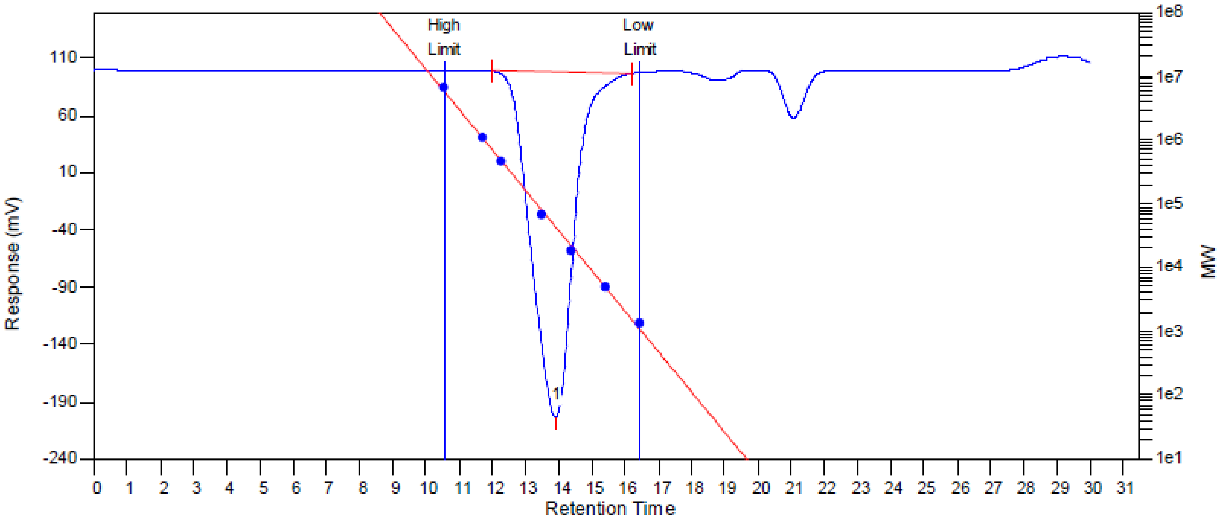

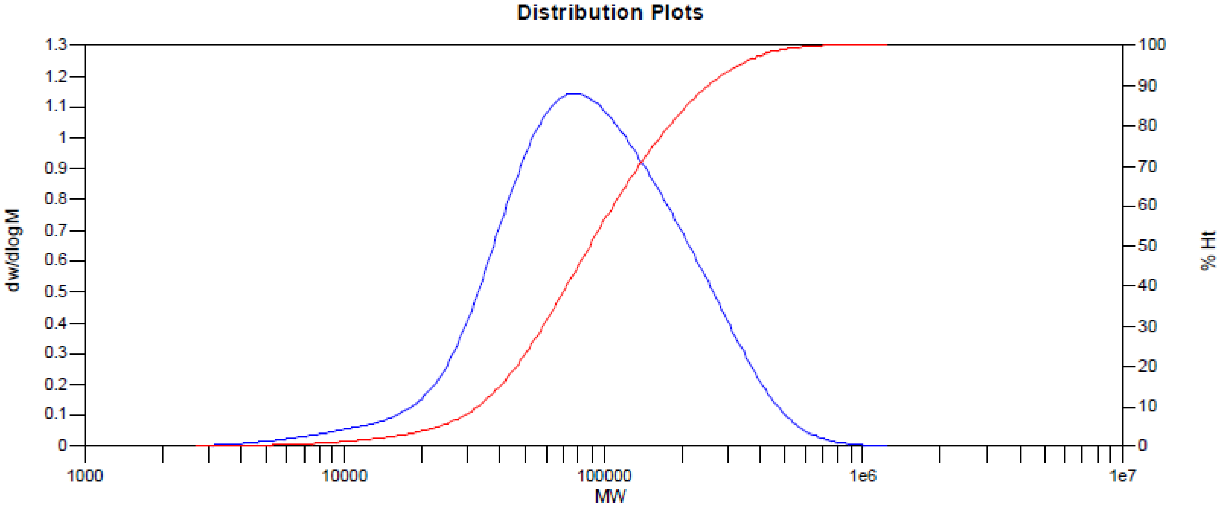

MW Averages

Peak No	Mp	Mn	Mw	Mz	Mz+1	Mv	PD
1	76319	60526	119615	207352	316156	108960	1.97626

Processed Peaks

Peak No	Name	Start RT (mins)	Max RT (mins)	End RT (mins)	Pk Height (mV)	% Height	Area (mV.secs)	% Area
1		12.02	13.92	16.20	-301.626	0	24860	100

Figure S74. GPC of polymer from table 2, entry 2

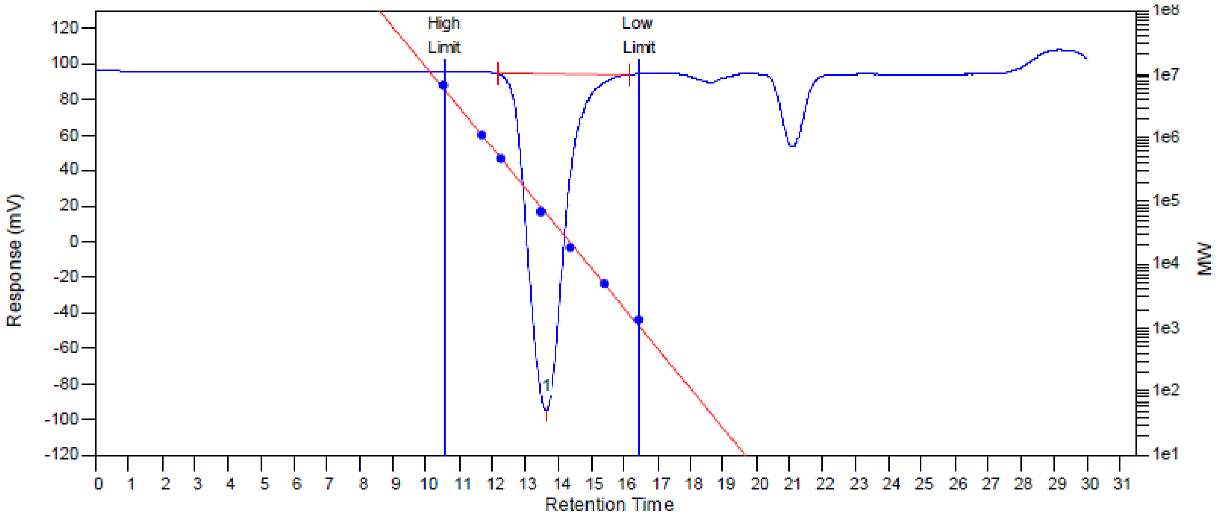

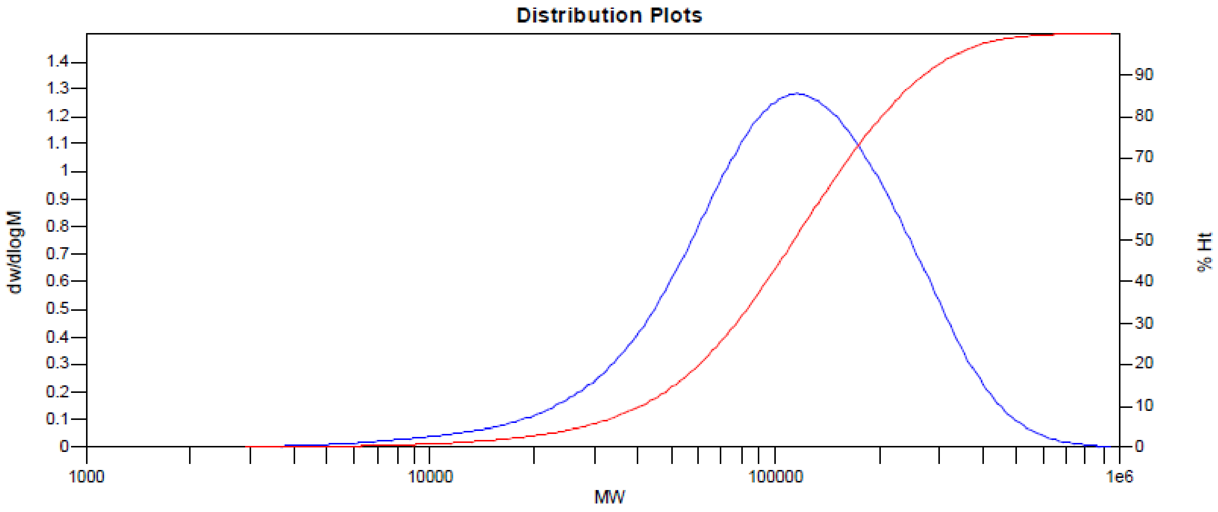

MW Averages

Peak No	Mp	Mn	Mw	Mz	Mz+1	Mv	PD
1	115583	75129	137043	208278	287412	127366	1.8241

Processed Peaks

Peak No	Name	Start RT (mins)	Max RT (mins)	End RT (mins)	Pk Height (mV)	% Height	Area (mV.secs)	% Area
1		12.20	13.63	16.15	-189.344	0	13934.9	100

Figure S75. GPC of polymer from table 2, entry 3

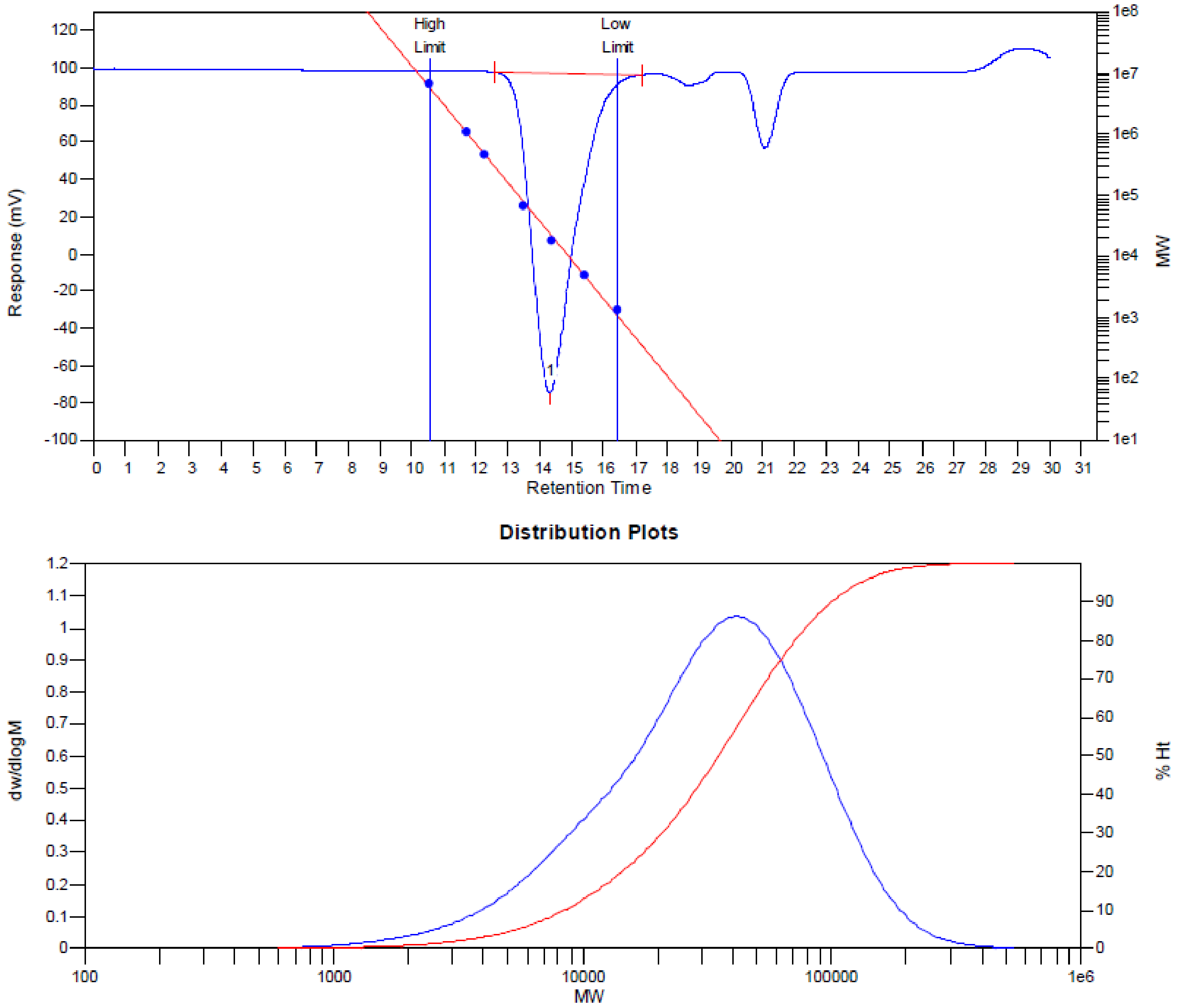

MW Averages

Peak No	Mp	Mn	Mw	Mz	Mz+1	Mv	PD
1	41452	18199	46426	85420	133667	41564	2.55102

Processed Peaks

Peak No	Name	Start RT (mins)	Max RT (mins)	End RT (mins)	Pk Height (mV)	% Height	Area (mV.secs)	% Area
1		12.58	14.33	17.22	-171.651	0	15646.5	100

Figure S76. GPC of polymer from table 2, entry 4

5. X-Ray Crystallography of the Palladium Catalysts

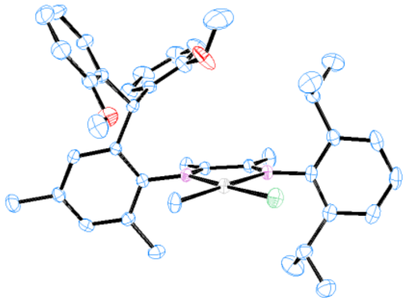

Crystal data structural refinement for Pd1

Formula	$C_{40}H_{49}ClN_2O_2Pd$
Formula Weight	731.66
Temperature/K	240
Crystal System	Monoclinic
Space group	P 1 21 1 (4)
a[Å]	8.3159(4)
b[Å]	23.0064(11)
c[Å]	9.9775(7)
α[°]	90
β[°]	108.177(2)
γ[°]	90
Volume [Å^3]	1813.63(18)
Z	2
D(calc)[g.cm^3]	1.340
μ [mm^{-1}]	0.621
F(000)	264.0
Radiations	MoKα (λ= 0.71073)
Θ min-max(°)	0.894-0.928
h	10
k	28
l	12
Reflection collected	7461 (3828)
Reflection unique	6797
Data completeness	1.78/0.91
GOF on F^2	1.074

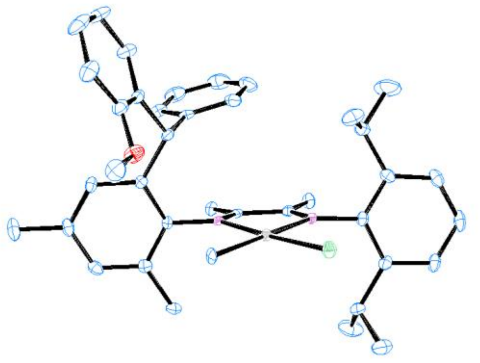

Crystal data structural refinement for Pd2

Formula	$C_{39}H_{47}ClN_2O_2Pd$
Formula Weight	701.63
Temperature/K	173
Crystal System	Triclinic
Space group	P -1 (2)
a[Å]	8.0184(6)
b[Å]	14.7016(11)
c[Å]	15.9540(12)
α[°]	104.177(3)
β[°]	101.120(3)
γ[°]	98.433(3)
Volume [Å^3]	1751.6(2)
Z	2
D(calc)[g.cm^3]	1.330
μ [mm^{-1}]	0.638
F(000)	732.0
Radiations	MoKα (λ= 0.71073)
Θ min-max(°)	2.64-26.07
h	9
k	18
l	19
Reflection collected	6933
Reflection unique	6667
Data completeness	0.962
GOF on F^2	1.086

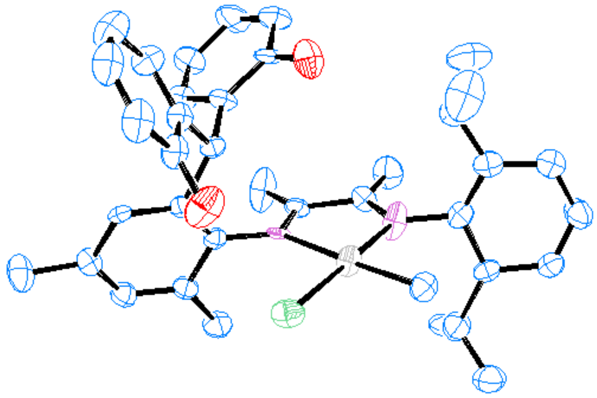

Crystal data structural refinement for Pd3

Formula	$C_{40}H_{49}ClN_2O_2Pd$
Formula Weight	703.61
Temperature/K	298
Crystal System	Orthorhombic
Space group	P c a 21 (29)
a[Å]	48.480(14)
b[Å]	8.274(2)
c[Å]	17.721(5)
α[°]	90
β[°]	90
γ[°]	90
Volume [Å^3]	7108(4)
Z	8
D(calc)[g.cm^3]	1.315
μ [mm^{-1}]	0.631
F(000)	2928.0
Radiations	MoKα (λ= 0.71073)
Θ min-max(°)	0.963-0.987
h	56
k	9
l	20
Reflection collected	11726 (6079)
Reflection unique	10451
Data completeness	1.72/0.89
GOF on F^2	1.103

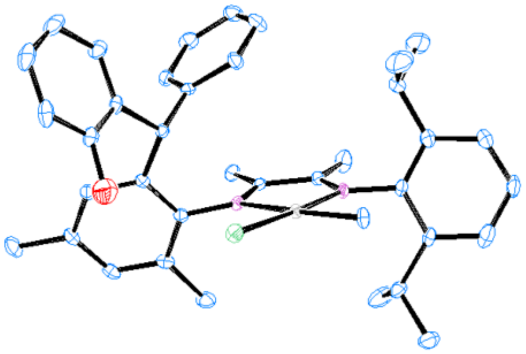

Crystal data structural refinement for Pd4

Formula	$C_{38}H_{45}ClN_2O_2Pd$
Formula Weight	687.61
Temperature/K	173
Crystal System	Triclinic
Space group	P -1 (1)
a[Å]	8.1638(10)
b[Å]	9.2154(11)
c[Å]	12.5641(15)
α[°]	105.094(4)
β[°]	101.120(3)
γ[°]	95.921(4)
Volume [$Å^3$]	106.342(4)
Z	1
D(calc)[g.cm^3]	1.328
μ [mm^{-1}]	0.648
F(000)	358.0
Radiations	MoKα (λ= 0.71073)
Θ min-max(°)	0.878,0.878
h	9
k	10
l	14
Reflection collected	5024[2512]
Reflection unique	4948
Data completeness	1.97-0.98
GOF on F^2	1.090
